# Monoallelic gene expression in developing cells increases genetic noise and Shannon entropy

**DOI:** 10.1038/s42003-025-08128-2

**Published:** 2025-06-04

**Authors:** Robert Wolff, Edoardo Balzani, Elisa Gelli, Alessia Polito, Angelo Serani, Valter Tucci

**Affiliations:** 1 Genetics and Epigenetics of Behavior Laboratory, Fondazione Istituto Italiano di Tecnologia (IIT), Genoa, Italy; 2 Padova Neuroscience Center (PNC), University of Padova, Padua, Italy; 3 Present Address: Center for Computational Neuroscience, Flatiron Institute, Simons Foundation, New York, NY USA

**Keywords:** Gene expression, Development, Imprinting

## Abstract

Monoallelic gene expression is a pivotal phenomenon in developmental biology, notably through the influence of imprinted genes. Our model predicts that monoallelic expression generates expression variability, which we assess by measuring genetic noise and entropy within Shannon’s information theory framework. Analyzing single-cell allele-specific expression across human and mouse datasets, we consistently observe increased expression variability due to monoallelic expression, affecting both imprinted and co-expressed non-imprinted genes. Moreover, we find decreasing variability in developing neurons and increasing variability in glial cells. The discovery of distinct noise patterns in over 80% of analyzed genes between glial and neuronal populations highlights the importance of differential noise in neurodevelopmental processes. Given the critical role of imprinted genes in biological processes such as growth and brain development, disruptions in their expression might contribute to various disorders. Understanding the stochastic nature of monoallelic expression and its genome-wide impact offers new insights into the mechanisms underlying these pathologies.

## Abstract (web summary)

The authors investigate the influence of genomic imprinting on genetic noise using complementary approaches. They estimate the expected increase in genetic noise associated with monoallelic expression by employing modelling and simulation techniques.

## Introduction

Genomic imprinting is an epigenetically regulated phenomenon that sets the allelic expression of certain genes within eukaryotic cells. In mammals, it exerts pivotal functions in embryogenesis, with consequences for the developmental programs of both high-complex cellular systems, such as neuronal networks, and cell-autonomous processes, such as the auto-regulatory cellular clock^1^. Imprinting controls the state of the cells through non-DNA post-mitotic heritable information in cell division^2,3^. Permissive and suppressive regulatory mechanisms coordinate the variation in allelic expression. Although this variation serves as an unavoidable source of noise for the cell^4–6^ our understanding of whether monoallelic expression influences the variation in gene expression remains limited. All biochemical events involved in regulating the abundance of gene products inherently exhibit stochasticity^7^. Hence, imbalances in allelic expression of imprinted genes can give rise to non-genetic stochasticity in cellular and network regulation, referred to as genetic noise. It disrupts precise genetic programs and interferes with cell signaling responses. However, genetic noise can also have advantages in certain processes, such as facilitating phenotypic diversification, probabilistic differentiation, state switching, excitability, and coordinated control of target genes, ensuring an evolutionary advantage, particularly in environments with stringent conditions^8^. Under specific conditions, populations consisting of haploid organisms, which exhibit monoallelic expression of all genes, may adapt faster compared to their diploid counterparts^9^.

Measuring genetic noise had been historically challenging until recent technological advances in single-cell RNA sequencing (scRNA-seq) allowed the study of gene expression at the cellular level. In this study we sought to investigate the influence of genomic imprinting on genetic noise using complementary approaches. Firstly, we performed a re-analysis of publicly available human and mouse scRNA-seq datasets^10–13^. These datasets allowed us to identify cell populations exhibiting a spectrum of expression patterns ranging from bi- to monoallelic expression of specific imprinted genes. By examining the impact of monoallelic expression on the genetic noise of both imprinted genes and their co-expressed genes, we gained insights into the effects of monoallelic expression on overall genetic noise. Secondly, we employed modeling and simulation techniques to study stochastic gene expression under two conditions: bi- and monoallelic gene expression. Through these simulations, we estimated the expected increase in genetic noise associated with monoallelic expression and explored how this increase in genetic noise is transferred to expressed and repressed genes. Thirdly, we introduced genetic entropy leveraging Shannon’s information theory and studied how it changes from bi- to monoallelic expression. Further, we investigated changes in mutual information between pairs of genes during the development of neurons and astrocytes in the mouse brain.

## Results

### Genetic noise and genetic entropy represent distinct aspects of gene expression dynamics

**Fig. 1:**
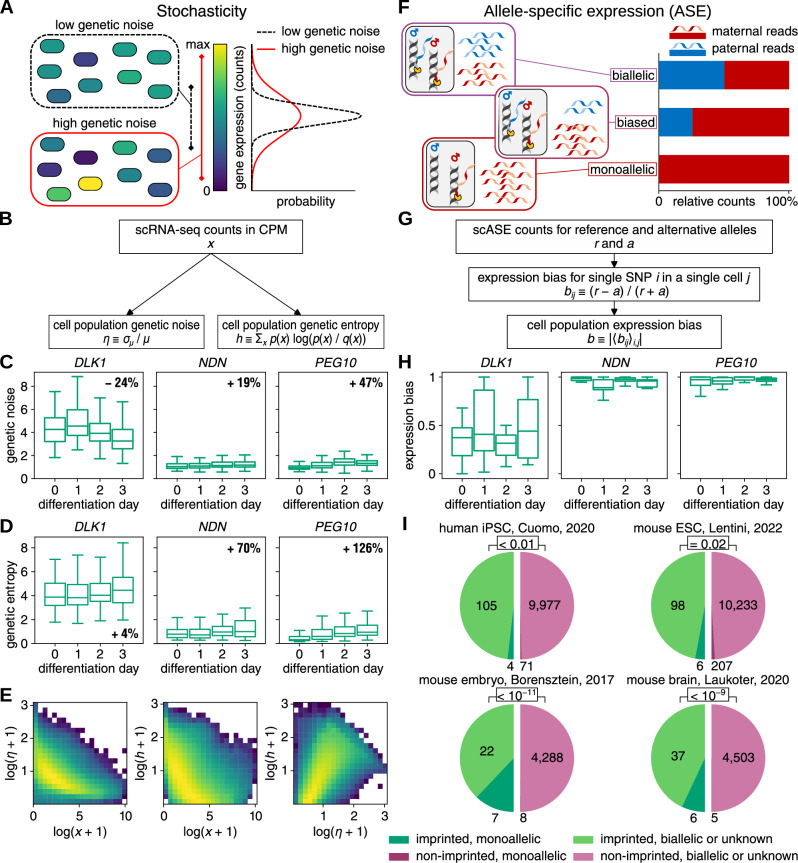
Genetic noise, entropy and expression bias. **A** Stochasticity in a cell population is described by the width of the gene expression distribution. **B** The scRNA-seq counts for the cells in a cell population are used to calculate the genetic noise and the genetic entropy of a given gene in a cell population. **C** Genetic noise of examples of imprinted genes in dependency of the day of differentiation in human-iPSC data. *N* = 401, 504 and 504 cell populations at different days of differentiation for *DLK1*, *NDN* and *PEG10*, respectively. **D** Genetic entropy for the same genes as in (**C**). **E** Dependency of genetic noise (left, *N* = 5,698,186 genes and cell populations) and genetic entropy (middle, *N* = 5,659,869) on the gene expression and the correlation between them (right, *N* = 5,611,818). **F** Biallelic, monoallelic or biased gene expression in single cells lead to different relative contributions of reads from maternal and paternal alleles. **G** The scASE counts for the reference and the alternative alleles are used to calculate the expression bias of a given gene in a cell population. **H** Expression bias of the same genes as in (**C**) in dependency of the day of differentiation in human-iPSC data. The value of the expression bias ranges from 0 for fully biallelic to 1 for fully monoallelic expression. *NDN* and *PEG10* are found with monoallelic expression. *N* = 47, 47 and 106 cell populations at different days of differentiation for *DLK1*, *NDN* and *PEG10*, respectively. **I** Monoallelic expression of imprinted and non-imprinted genes in four datasets for genes with at least 5 cell populations after quality assessment for bias calculation. *p*-values were calculated using the exact Fisher test. **C**, **D** and **H** In the box plots, the box extends from the first to the third quartile, with a line at the median, and the whiskers extend to the farthest data points lying within 1.5 times the interquartile range.

We analyzed gene expression data obtained from human induced pluripotent stem cells (iPSCs) differentiated into endoderm, which provided single-cell allele-specific expression (scASE) levels^11^. The data includes 11,231 genes, expressed in 36,044 cells across four developmental stages (from day 0 undifferentiated to day 3 post-differentiation initiation). We matched 115 of these genes to known imprinted genes. We conducted a principal component analysis and *k*-means clustering to match cells from different differentiation stages to well-separated clusters (Fig. S1–1, see Supplemental Methods). We removed 3,765 cells that did not match the associated clusters, due to delayed (50%) or failed (25%) differentiation (Fig. S1–1D). The scRNA-seq counts for the 32,279 remaining cells were used to estimate the variability of the expression of a gene in a cell population (Fig. 1A, B). In previous studies, various metrics of variability, such as the Fano factor or distance to median, have been employed^14,15^. In our research we adopted the conventional definition of genetic noise as the coefficient of variation. Additionally, we propose genetic entropy using the Kullback–Leibler divergence, also known as relative entropy. It measures how divergent the distribution of gene expression in a cell population is from the overall gene expression distribution for a gene. Both genetic noise and genetic entropy assess different aspects of variability. The data comprise 517 cell populations, with each population representing cells obtained from a single donor and differentiated on a specific experimental dish on a particular day of the differentiation process. For a given gene, we discarded cell populations with a gene expression of *μ* < 0.2 counts per million (CPM) to reduce artificially high genetic noise at low counts (Fig. S1–2A). In Fig. 1C, it is evident that the genetic noise of specific imprinted genes exhibits a correlation with the day of differentiation. For instance, the gene *PEG10* demonstrates a 47% increase in genetic noise throughout the differentiation process. This observed increase could arise from intrinsic or extrinsic factors^16,17^. The differential gene expression observed during differentiation can serve as a potential source of intrinsic genetic noise, which naturally increases at low levels of molecules. In addition, cell populations diverge during differentiation, which represents a source of extrinsic genetic noise. We separated the change in genetic noise during differentiation from that caused by monoallelic gene expression in our analysis. Similarly, the genetic entropy is correlated with the day of differentiation, such as the genetic entropy of *PEG10* increases by 126% during differentiation (Fig. 1D). As expected, we found that higher gene expression leads to lower genetic noise. A similar relation is seen for the dependency of genetic entropy on gene expression. Genetic noise and genetic entropy are positively correlated but measure distinct aspects of gene expression variability (Fig. 1E).

Analyzing the correlation of gene expression and genetic noise of pairs of imprinted genes in the whole genome, within the same chromosome, and in the same locus, we observed no difference in the correlation between imprinted gene pairs from the same or from different chromosomes. However, we found that pairs of imprinted genes regulated by the same imprinting control region have increased correlation. For instance, the imprinted genes *UBE3A*, *SNRPN*, *NDN* and *SNURF* in the SNURF-SNRPN locus exhibited high correlation of gene expression and genetic noise (Fig. S1–2D, E).

### Monoallelic expression is primarily observed for imprinted genes

To assess the expression pattern of genes within a cell population, we investigated whether they displayed biallelic or monoallelic expression. This analysis was made possible by leveraging the single-nucleotide variations between maternal and paternal alleles in the human genome (Fig. 1F). For each single-nucleotide polymorphism (SNP), we utilized the scASE counts of the reference and alternative alleles to compute the single-SNP single-cell expression bias, denoted as *b*_*ij*_. We then defined the expression bias *b* of a gene in a cell population by custom averaging of *b*_*ij*_ over the SNPs matched to that gene and over the cells in the cell population (Fig. 1G, see Supplemental Methods). Thus, it ranges from 0 for fully biallelic to 1 for fully monoallelic expression. Cell populations were filtered by certain quality cuts to increase the reliability of the expression bias estimate (Fig. S1–3A, B). In the example of *DLK1*, 31 of the 47 cell populations have an expression bias *b* ≈ 0, meaning that *DLK1* is mostly biallelically expressed. However, there are also 16 cell populations with an estimated expression bias *b* ≥ 0.5, corresponding to a monoallelic expression (Fig. 1H). On the other hand, the genes *NDN* and *PEG10* are monoallelically expressed during the whole differentiation (Fig. 1H). Out of the total 115 imprinted genes, we observed that *NDN*, *PEG10*, *SNRPN*, and *SNURF*, which constitute 4% of all imprinted genes, were expressed in a monoallelic manner in the cells. We identified 71 non-imprinted genes (1% of all) in this context. Among 47 genes that are predicted to be imprinted, only *IFITM1* showed strong monoallelic expression (Fig. 1I and S1–4, Methods). However, we did not observe any imprinted gene with a significant dependence of the expression bias on the day of differentiation. We conducted an analysis of three scRNA-seq datasets obtained from mice. The first dataset encompassed the differentiation process of embryonic stem cells (ESCs) into epiblast stem cells, consisting of 3412 cells^13^. The second dataset covered the embryonic developmental stages from oocytes to blastocysts, comprising 113 cells^10^. The third dataset consists of 404 cells from the developing brain at birth and at postnatal day 42^12^. These studies utilized mouse lines generated by crosses between C57BL/6J and CAST/EiJ strains. Given the presence of abundant SNPs and knowledge of the mouse origin, we estimated maternal and paternal gene expression using the scASE data. In the mouse-embryo and human-iPSC data we found that imprinted genes exhibit higher genetic noise than non-imprinted genes, while in the mouse-ESC and in the mouse-brain data, we observed comparable levels of genetic noise between imprinted and non-imprinted genes (Fig. S1–5). In the mouse-ESC data 6 (6%) imprinted and 207 (2%) non-imprinted genes have monoallelic expression (Fig. 1I and S1–6). In the mouse-embryo data 7 (24%) imprinted and 8 (0.002%) non-imprinted genes are rather monoallelically expressed (Fig. 1I and S1–7). Similarly, in the mouse-brain data we observed 6 (14%) imprinted and 5 (0.001%) non-imprinted genes with monoallelic expression (Fig. 1I and S1–8). Notably, certain genes that demonstrated monoallelic expression during embryonic development exhibited a more biallelic expression pattern during ESC differentiation (Fig. S1–9B). These findings suggest that imprinting may be partially lost or not yet fully established during human iPSC and mouse ESC differentiation^18,19^, leading to an increased prevalence of monoallelic expression in non-imprinted genes. Furthermore, stable random monoallelic expression has been previously reported for clonal cell populations^20,21^. Nonetheless, across all four datasets, we consistently observed a higher proportion of imprinted genes exhibiting monoallelic expression compared to non-imprinted genes (Fig. S1–5E).

### Monoallelic expression amplifies variability in gene expression

**Fig. 2:**
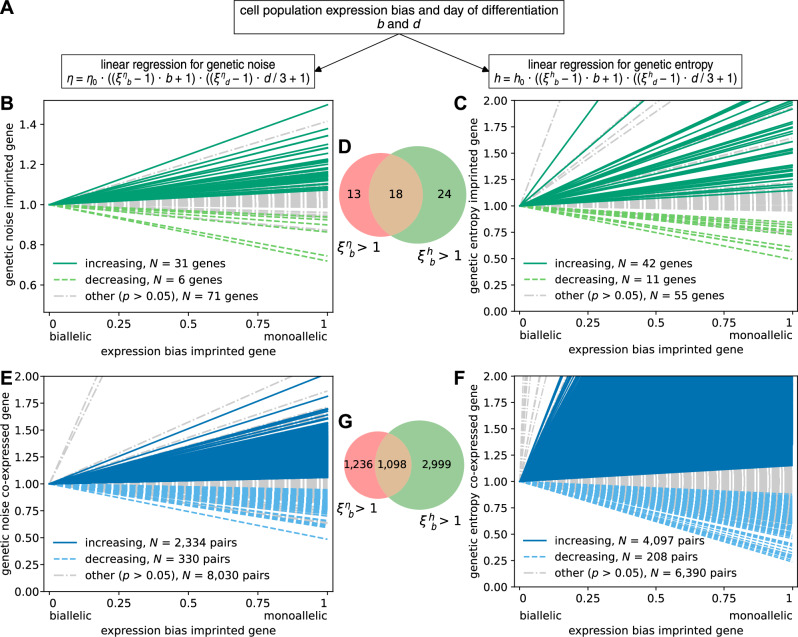
Genetic noise and genetic entropy are increased at monoallelic expression in human-iPSC data. **A** For each gene both the expression bias and the day of differentiation are used in the multiple linear regression for genetic noise and genetic entropy. Normalized genetic noise (**B**) and genetic entropy (**C**) of *N* = 108 imprinted genes with at least 10 cell populations in dependency of their expression bias, estimated by linear regressions. Of these, 31 have an increase (*p* < 0.05) in genetic noise and only 6 have a decrease (*p* < 0.05). Similarly, 42 have an increase in genetic entropy and 11 have a decrease. **D** Venn diagram of imprinted genes with increase in genetic noise (left) and genetic entropy (right). Genetic noise (**E**) and genetic entropy (**F**) of all co-expressed genes of 58 imprinted genes with at least 10 cell populations and co-expression clusters available. **G** Venn diagram of pairs of imprinted and co-expressed genes with increase in genetic noise (left) and genetic entropy (right).

We used the estimates of genetic noise, genetic entropy, and expression bias for cell populations during human endoderm differentiation to examine how variability in gene expression depends on the number of expressed alleles. We conducted a multiple linear regression to estimate the degree of dependence of genetic noise or genetic entropy on both expression bias and the day of differentiation simultaneously (Fig. 2A). We defined the genetic noise change from bi- to monoallelic expression (*ξ*^*η*^_*b*_) as the ratio of genetic noise values at expression bias *b* = 1 to *b* = 0, such that a value greater or less than 1 corresponds to an increase or a decrease in genetic noise, respectively. Similarly, the genetic noise change during differentiation (*ξ*^*η*^_*d*_) is the ratio of genetic noise values at day 3 to day 0 of differentiation. The genetic entropy change (*ξ*^*h*^_*b*_ and *ξ*^*h*^_*d*_) is analogously defined (see Supplemental Methods).

108 of 115 imprinted genes are expressed with scASE counts in at least 10 cell populations and were considered in our analysis. We found 31 imprinted genes with a genetic noise increase from bi- to monoallelic expression (*p* < 0.05, Fig. 2B and S2–3A, Supplementary Data 2). 42 imprinted genes demonstrate a genetic entropy increase at monoallelic expression (Fig. 2C, Supplementary Data 3). In consistency with the correlation of genetic noise and genetic entropy reported in Fig. 1E, 18 imprinted genes have an increase in both genetic noise and genetic entropy at monoallelic expression (Fig. 2D). We investigated the gene ontology comparing the distinct groups of genes and did not find major enhanced terms. To study the impact of gene expression variability of an imprinted gene on the gene expression variability of other genes we examined the co-expression clusters (see Methods). We found that at monoallelic expression of imprinted genes 73% of their co-expressed genes (22% with *p* < 0.05) have increased genetic noise (Fig. 2E, Supplementary Data 4). Similarly, 84% of co-expressed genes (38% with *p* < 0.05) show an increase in genetic entropy (Fig. 2F, Supplementary Data 5). 1,098 pairs of imprinted and co-expressed genes have both increasing genetic noise and genetic entropy at monoallelic expression of the imprinted gene (Fig. 2G). It is important to emphasize that co-expression between two genes does not directly indicate causality. Consequently, our co-expression analysis does not determine whether imprinted genes enhance the expression of their co-expressed genes, such as through transcriptional activation, or if the observed correlation in gene expression is due to other underlying factors. However, we observed an increased genetic noise in most co-expressed genes when the imprinted genes exhibited monoallelic expression. This suggests a permissive effect of the imprinted genes on most co-expressed genes. The observed increase in genetic noise and genetic entropy at monoallelic expression is also observed for non-imprinted genes (Fig. S2–2A–C, Supplementary Data 6, 7), suggesting that it is an effect of the monoallelic expression itself.

We analyzed genetic noise during development in the three mouse scRNA-seq datasets. To address the issue of low statistics (Fig. S2–9A), we devised an overall analysis method that combines data from all genes and cell populations to estimate the relationship between genetic noise and expression bias. We applied this overall analysis to the human iPSC data and obtained results consistent with the previous detailed analysis, with an overall increase of 5% and 22% in genetic noise during monoallelic expression and differentiation, respectively (Fig. S2–9B). In the mouse ESC data, we observed an overall increase of 38% and 40% for maternal and paternal monoallelic expression, respectively (Fig. S2–9C). Similarly, the mouse embryonic development data showed an overall increase of 14% and 22% for maternal and paternal expression, respectively (Fig. S2–9D). Given the potential variations in genetic noise levels among distinct cell types, particularly during brain development, we conducted separate analyses for astrocytes and neurons in the mouse brain data. These separate analyses revealed an increase in genetic noise linked to monoallelic expression; however, we observed that during development (from postnatal day P0 to P42), the genetic noise increases for astrocytes (from astrocyte progenitor cells at P0 to astrocytes at P42) whereas it decreases for neurons (Fig. S2–10). We investigated the biological functions of the four classes of genes characterized by genetic noise increases or decreases in astrocytes or neurons (Fig. S2–11B, C). For instance, genes exhibiting high genetic noise in developed neurons were associated with signaling pathways. Conversely, genes displaying high genetic noise in developed astrocytes were linked to cell cycle and proliferation pathways, as well as transcription, translation, and catabolism pathways. Genes with low genetic noise at P42 for both neurons and astrocytes exhibited enrichment in pathways related to metabolism.

### Dosage compensation accompanies monoallelic expression both in imprinted and non-imprinted genes

To test if the effects on genetic noise and genetic entropy were only due to mRNA abundance, we checked whether imprinted genes were upregulated. If imprinting acts purely as dosage regulation, the gene expression for monoallelic expression is expected to be half compared to biallelic expression. In the case of full dosage compensation, the gene expression is the same for biallelic and monoallelic expression. In total, 98 of 104 imprinted genes (for 4 imprinted genes the linear regression did not succeed) have a gene expression change *ξ*^*μ*^_*b*_ > 0.5 (Fig. S2–6 and S2–3B, Supplementary Data 8). The observed median gene expression change for all 104 imprinted genes is *ξ̄*^*μ*^_*b*_ = 0.90, consistent with the expectation of 1 for dosage compensation (Fig. S2–1C, D, Supplementary Data 8). We also investigated the gene expression of the non-imprinted genes and found similar dosage compensation at monoallelic expression as of the imprinted genes (Fig. S2–1C, D, S2–2D and S2–3B, Supplementary Data 9). These data confirm previous evidence that imprinted genes show upregulation^22^.

### Genetic noise is independent of the parental origin of the expressed allele and of the sex of the donor

Genomic imprinting involves parent-of-origin mechanisms. During gamete formation, genes are marked or imprinted, resulting in distinct gene expression patterns based on their parental origin. This non-random process plays a critical role in various biological processes and development. Consequently, disruption of specific alleles in this process can contribute to the development of genetic disorders. We hypothesized that the increase in genetic noise would not be influenced by whether the expressed allele is inherited from the mother or the father. Both maternal and paternal monoallelic expression lead to the expression of a single allele. To test this hypothesis, we conducted linear regressions where genetic noise depended on the average expression bias, rather than on the absolute value. We found no difference in the genetic noise change for monoallelic and inversely monoallelic expression, suggesting that the genetic noise increase is not a parent-of-origin effect (Fig. S2–7A, B, Supplementary Data 10). Another way to investigate the parent-of-origin effect is the use of previous knowledge about the imprinted genes. Maternal and paternal imprinting is reported for 63 and 28 of the 108 imprinted genes, respectively (see Methods). We observed no difference between maternally and paternally imprinted genes. Further, we investigated the dependency of the genetic noise change on the sex of the iPSC donor. Overall, we found no dependency of the genetic noise change on the sex of the donor (Fig. S2–7C, D, Supplementary Data 11).

### Intrinsic and extrinsic factors contribute to increased gene expression variation during differentiation in imprinted and non-imprinted genes

In the human-iPSC data, genetic noise increases during differentiation, with a notable rise observed from day 1 to day 2 of the differentiation process (Fig. S1–2B). The median estimated increase in genetic noise for imprinted genes during differentiation is 17%, and we observed an increase in noise for 75% (67% with *p* < 0.05) of imprinted genes (Fig. S2–8A and S2–3B). The correlation between genetic noise change and gene expression change is attributed to smaller molecule numbers resulting in higher intrinsic noise. We found a linear relationship between the logarithm of noise change and the logarithm of expression change (Fig. S2–8A). By utilizing the slope of this regression, we separated the extrinsic noise change caused by differentiation from the intrinsic noise change resulting from differential gene expression. Our observations indicate that the median extrinsic noise increases by 20%, while the median intrinsic noise remains unchanged (Fig. S2–8B and S2–1E). Similarly, non-imprinted genes show a 20% increase in extrinsic noise and a 1% increase in intrinsic noise (Fig. S2–1E and S2–2E, F). Overall, our findings demonstrate that the genetic noise of both imprinted and non-imprinted genes increases during differentiation due to contributions from both intrinsic and extrinsic factors.

### Simulating monoallelic expression and genetic noise: implications for imprinted genes and gene networks

We conducted simulations to model gene expression based on a mathematical approach inspired by classical eukaryotic gene expression studies^17,23^. The simulation involved various molecular components and processes (Fig. S2–12A). Specifically, we incorporated permissive and repressive interactions of an imprinted gene with other genes (Fig. S2–12B). In this model, the activation or inactivation rate of the expressed or repressed gene was modulated by the protein count of the imprinted gene. Thus, the imprinted gene acted as a transcription factor that positively regulated the expressed gene and inactivated the DNA of the repressed gene. We simulated biallelic expression with a maximum number of two alleles that can be activated. To simulate monoallelic expression, we allowed only one allele to be activated, which would lead to a halved protein count and to compensate for the dosage, we doubled the mRNA transcription rate. This allowed us to estimate the genetic noise change solely due to monoallelic expression. By running simulations with different sets of process rates for the imprinted gene, we observed an increase in genetic noise from biallelic to monoallelic expression. Additionally, we observed a transfer of genetic noise to the expressed and repressed genes (Fig. S2–12C). The estimated genetic noise increase for the imprinted gene during the transition from biallelic to monoallelic expression was 36%. In comparison, the observed median increase in genetic noise from the human-iPSC data was 17% for imprinted genes with increased genetic noise, indicating a smaller increase (Fig. S2–12D). However, it is important to note that our simulated model simplifies the gene expression process, and the actual genetic networks and additional regulatory mechanisms could further influence genetic noise. The genetic noise of the expressed genes showed an increase of 25%, which was smaller than the increase observed in imprinted genes, like observed in the human-iPSC data, where the genetic noise of co-expressed genes increased by 6% (Fig. S2–12E). The observed difference may be explained by the simplifications of the simulated model, such as imprinted genes acting as transcription factors to their co-expressed genes, and the limitations of the presented analysis, which did not measure the causality in correlated expression. We did not measure the genetic noise change of repressed genes in the human-iPSC data. In our simulations, we estimated a mean genetic noise increase of 28%, but with high variability depending on the process rates (Fig. S2–13). Overall, our simulation results align with the measured genetic noise increase values for imprinted genes and their co-expressed genes. Furthermore, these findings suggest that the genetic noise is also transferred to repressed genes.

### Mutual information in mouse-brain data: how information transfer changes during development

**Fig. 3:**
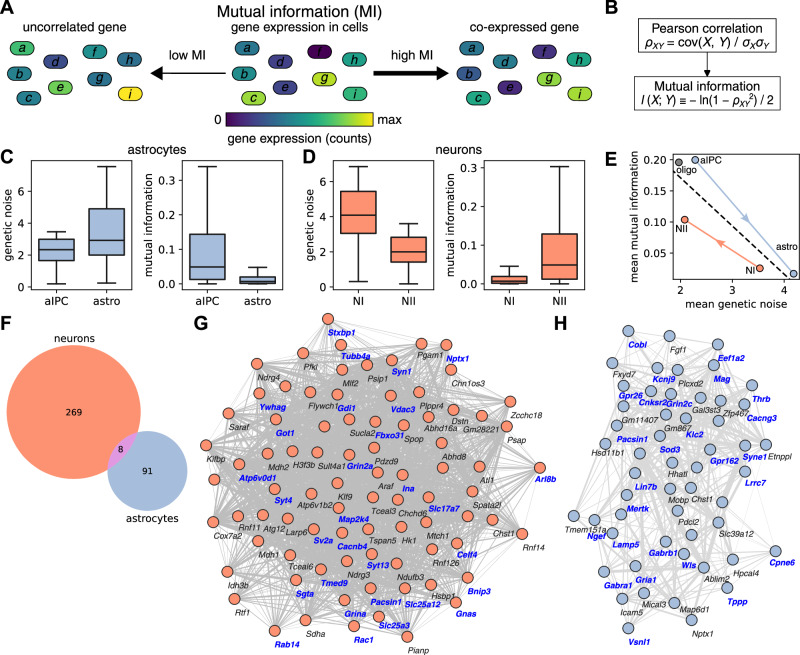
Mutual information (MI) in the mouse brain data. **A** The MI between two genes depends on the correlation of gene expression of these genes. Thus, uncorrelated genes have low and co-expressed genes have high MI. **B** The Pearson correlation is used to estimate the MI. Genetic noise of *N* ≈ 190 thousand genes and MI of *N* ≈ 360 million pairs of genes for astrocyte (**C**) and neuron (**D**) cell populations during development. aIPC: astrocyte intermediate progenitor cells, astro: mature astrocytes, NI: nascent projection neurons, NII: mature projection neurons. **E** Genetic noise and MI, as shown in (**C**) and (**D**), are inverse proportional to each other. Additonally, the mean genetic noise and MI of oligodendrocytes (oligo) is shown. **F** Genes with the highest (more than two times median) change in MI for astrocytes and neurons during development have little overlap. **G** Network of genes with the highest change in MI for neurons. *N* = 83 genes are shown for which the node’s degree (number of edges with other genes with high MI) is greater than 58. Genes in blue bold font contribute to enriched gene ontology terms related to brain functioning. **H** Network of genes with the highest change in MI for astrocytes. *N* = 53 genes are shown for which the node’s degree is greater than 15. **C** and **D** In the box plots, the box extends from the first to the third quartile, with a line at the median, and the whiskers extend to the farthest data points lying within 1.5 times the interquartile range.

We investigated the mutual information between pairs of genes in cell populations from mouse-brain data. Two genes that are co-expressed or that have a high correlation have a high mutual information (Fig. 3A). We estimated the mutual information using the Pearson correlation (Fig. 3B). During development from astrocyte intermediate progenitor cells (aIPCs) at post-natal day P0 to astrocytes at P42, the genetic noise of genes increases, while the mutual information between pairs of genes decreases (Fig. 3C and S2–10A). On the other hand, developing neurons from NI at P0 to NII at P42 show an opposite trend of decreasing genetic noise and increasing mutual information (Fig. 3D and S2–10B). Genetic noise and mutual information are found inversely proportional to each other (Fig. 3E, including the data on oligodendrocytes). We investigated which genes are the ones with the highest change in mutual information with other genes. In astrocyte development, 99 genes have a mean mutual information decrease greater than two times the median. During neuron development, 279 genes exhibit a strong increase in mutual information with other genes. The genes with big mutual information change are different for astrocyte and neuron development (Fig. 3F). Gene ontology (GO) study of these genes against the background of all other genes shows enrichment in many GO terms related to brain functioning. For developing neurons, more than one-third of the genes with big mutual information change contribute to enriched GO terms such as synaptic signaling, ion channel activity and various neuronal cell components (Fig. 3G). In the case of astrocyte development, channel activity, signaling receptor activity and various cell membrane components are enriched GO terms, associated to circa one half of the genes with big mutual information change (Fig. 3H). Further, we investigated if the expression bias of imprinted or non-imprinted genes changed during development. We did not observe such a change, but the study was limited by low cell and read numbers. This is consistent with previous findings by Laukoter et al. for selected imprinted genes^12^.

## Discussion

One of the key evolutionary strategies employed in genome construction involves the creation of surplus copies of chromosomes, a phenomenon seen in diploidy or polyploidy, followed by the silencing of some excess copies through mechanisms such as X-inactivation or imprinting. While the theoretical implications of imprinting can be complex and beyond the scope of our study, our hypothesis posited that imprinting, by reducing these surplus copies, introduces non-genetic stochastic variability. Previous attempts to propose imprinting as a mechanism for regulating transcriptional variation had suggested that it minimizes variance^24^. This idea was grounded in the assumption that imprinting evolved to select for reduced variation in gene expression levels. However, empirical evidence supporting this hypothesis was lacking. Our study now provides high-resolution theoretical and experimental evidence that contradicts this notion, revealing that imprinting increases genetic noise due to monoallelic expression. Furthermore, we demonstrated that this imprinting-dependent regulatory effect can extend to other genes and varies across cells and cellular states. Thus, imprinting in each individual cell emerges as a significant source of genetic noise, suggesting that imprinted genes can both generate and regulate genetic noise throughout the genome.

Monoallelic expression in iPSC lines has been previously documented^25^, as has the importance of imprinted genes in iPSC populations^26^. Whether a few or all somatic cells are susceptible to iPSC reprogramming, the presence of stochastic epigenetic events in acquiring pluripotency is well recognized^27^. We observed high variability in genetic noise and genetic entropy changes among imprinted genes and their co-expressed partners, possibly due to differences in gene expression rates. We also noted that genetic noise and genetic entropy of most imprinted genes increased as iPSCs differentiated into specialized cell types. This increase could be attributed to intrinsic noise stemming from decreased gene expression and extrinsic noise due to differentiation. Extrinsic sources of genetic noise include variations in cell states at different differentiation stages, with day 0 representing the most uniform, undifferentiated state. Monoallelic expression of imprinted genes contributes to intrinsic noise, and the transferred noise to expressed and repressed genes can be categorized as local extrinsic noise^16,17^.

Imprinting plays a crucial role in various developmental stages of an organism, possibly serving as a mechanism to maintain embryonic diploidy^28^. Increased variation in gene expression levels resulting from imprinting may significantly impact cellular phenotypic mechanisms. On an evolutionary scale, a similar phenomenon, known as ‘conflict-induced decanalization’, has been recognized as a mechanism for controlling phenotypic variation based on parental allele conflicts^29^.

Regarding the role of different alleles in variability in gene expression, we found that this effect is unrelated to the allele’s origin but instead associated with imprinted gene expression biases. Recent evidence also suggests that the parental origin of the epigenetic state of an imprinted gene is not relevant^30^, and we believe that the stochastic effect resulting from allelic silencing is independent of its parental origin. In recent years, many potentially imprinted genes have been proposed based on parent-of-origin expression biases. Of the 47 predicted imprinted genes we analyzed, only one was found to be monoallelic. Recent evidence suggests that most newly proposed imprinted genes exhibit weak expression bias, possibly due to regulation by flanking imprinting control regions or specific chromosome configurations in certain tissues or developmental stages^31^. These considerations, along with the limitations of scASE data, underscore the need for proper validation of novel imprinted genes. Moreover, our results suggest that biases towards one allele may be random.

Thus, it is reasonable to hypothesize that gene expression variability generated by imprinting throughout development could exert a noteworthy influence. Particularly during brain maturation, phenotypic variation among neural cells becomes critical for establishing brain circuits. In the perinatal period, the developing brain undergoes extensive production of neuronal elements, accompanied by competitive plasticity mechanisms and eventual pruning^32,33^. Numerous imprinted genes have been identified as key players in regulating various neurodevelopmental processes, including neurogenesis, migration, neurite growth, apoptosis, synaptic formation, transmission, and plasticity^34,35^.

Since Hubel and Wiesel’s seminal discovery almost six decades ago^36^, it has been recognized that changes in neuronal activity, both spontaneous and induced by sensory experiences, contribute to the rewiring of the developing brain. However, the precise molecular and epigenetic mechanisms underlying these brain processes have remained enigmatic. An integral aspect of central nervous system development in mammals, specifically synaptic pruning, involves the selective elimination of specific synapses while preserving others, with glial cells primarily regulating this pivotal process.

Our findings reveal a significant alteration in genetic noise and genetic entropy during a critical period characterized by essential brain maturation and specialization processes. For instance, we have observed an increase in genetic noise in genes associated with synaptic pruning, such as *Cdk5*, *Adgrb3*, and *Epha4*, in both astrocytes and neurons as cell maturation advances. One plausible reason for the increased gene expression variability observed in glial cells could be attributed to their complex roles, which include maintaining the structural integrity of the brain, supplying metabolic support to neurons, and overseeing the regulation of synaptic activity. These functions demand a greater level of adaptability. The high genetic noise may endow them with the capacity to swiftly respond to fluctuating environmental cues, enabling them to finely adjust their support for neurons based on real-time demands.

We conducted a more comprehensive analysis of a subset of these data utilizing stringent statistical criteria. This analysis revealed several targets linked to synapse formation, maturation, and plasticity. The enriched genetic noise in these cells could effectively represent the fine-tuning of synaptic connections during critical developmental phases and even during the dynamic processes of learning and memory. Moreover, our study has unveiled intriguing signals related to the regulation of the circadian clock, synaptic signaling and homeostasis, and RNA synthesis. Recent evidence underscores the significant role of imprinting in the regulation of circadian rhythms^37–39^. This is further substantiated by the presence of numerous imprinted genes enriched within the central master clock of the brain, the suprachiasmatic nucleus of the hypothalamus^40,41^. These findings align with the notion that glial cells may play a pivotal role in supporting neuronal connectivity and synchronization during brain development.

In conclusion, there is evidence that imprinted genes are part of regulatory networks influencing biological functions^34,42^ and exerting control over non-imprinted genes^43^. The functions of these networks may be altered by dosage variations in imprinted genes. Therefore, genomic imprinting will be instrumental to study polygenic effects on brain functions during development.

## Methods

### Definitions of observable quantities

In a cell population for a gene, the following parameters are defined: (1) The genetic noise is *η* = *σ* / *μ* with the standard deviation *σ* and mean *μ* of gene expression in the cell population. (2) The genetic entropy is *h* = Σ_*x*_ *p*(*x*) ⋅ log(*p*(*x*) / *q*(*x*)) where *p*(*x*) and *q*(*x*) are the distributions of gene expression in the cell population and all cells of all cell populations, respectively. (3) The expression bias is *b* = (*r* − *a*) / (*r* + *a*) with the scASE counts for reference *r* and alternative *a* allele (see Supplemental Methods). (4) The mutual information between two genes is defined using the Pearson correlation coefficient *ρ*_*XY*_ of their gene expression as *I*(*X*; *Y*) = − ln(1 − *ρ*_*XY*_^2^) / 2 (see Supplemental Methods).

### Quality assessment of cell populations

We performed principal component analysis and *k*-means clustering and kept only cells that have matching cluster to day of differentiation (see Supplemental Methods). To achieve a robust statistical analysis of a variable, several requirements were implemented: (1) For the calculation of genetic noise and genetic entropy, the gene expression of the investigated gene must satisfy *μ* ≥ 0.2 CPM. For all data points of cell populations and genes with scRNA-seq data available 97.3% pass this selection (Fig. S1–2A, B). (2) For the expression bias calculation, cell populations must have the bias information available for at least 5 cells with a total count of at least 25 reads. Out of 59,455 data points of 517 cell populations and 115 imprinted genes with scASE data available 35,419 have at least one cell with bias information available, and 22,839 pass these selection criteria (Fig. S1–3).

### Statistical analysis

To assess the quantitative change of a dependent variable on the expression bias *b* and the day of differentiation *d* for a given gene, we implemented a multiple linear regression, e.g., for the genetic noise with the parametrization *η* = *η*_0_ ⋅ ((*ξ*^*η*^_*b*_−1) ⋅ *b* + 1) ⋅ ((*ξ*^*η*^_*d*_−1) ⋅ *d*/3 + 1). Here, *η*_0_ is the genetic noise at differentiation day *d* = 0 and expression bias of *b* = 0 (biallelic expression), *ξ*^*η*^_*b*_ is the genetic noise change from biallelic to monoallelic expression, and *ξ*^*η*^_*d*_ is the genetic noise change from day 0 to day 3 of differentiation (see Supplemental Methods).

### Classification of imprinted, non-imprinted, and co-expressed genes

The 11,231 expressed genes in the human-iPSC data^11^ were categorized into 115 imprinted and 11,116 non-imprinted genes using prior knowledge. 100 genes were matched to imprinted genes in the Supplemental Table of a review on imprinting^1^ and additional 15 genes (*CHD2*, *DGCR6*, *DGCR6L*, *DIO3*, *GLI3*, *MAGI2*, *NLRP2*, *OSBPL5*, *PARD6G*, *PPP1R9A*, *RAC1*, *RBP5*, *ST8SIA1*, *TP53* and *ZNF396*) to imprinted genes reported by geneimprint (accessed on 16/12/2022). Further, 47 genes from geneimprint, which are predicted to be imprinted, were included in the set of non-imprinted genes in our analysis. Of the 115 imprinted genes, 66 are maternally and 32 paternally imprinted. Similarly, the 16,093 expressed genes in the mouse-ESC data^13^ were matched to 149 imprinted genes, of which 80 are maternally and 59 are paternally imprinted. The 15,253 expressed genes in the mouse embryo data^10^ were matched to 161 imprinted genes, with 82 maternally and 70 paternally imprinted genes. 60 clusters of co-expression have been published with the human-iPSC data^11^. Co-expression clusters were defined using affinity propagation with the Pearson correlation as an affinity metric. Co-expressed genes were available for 61 of 115 imprinted genes. 19 pairs of imprinted genes were found that are within the same imprinting locus^1^ with the following genes: *ACCS*, *ALKBH3* (ACCS locus); *SLC22A18*, *CDKN1C*, *ZNF215*, *IGF2*, *PHLDA2* (IGF2 locus); *PHACTR2*, *PLAGL1* (HYMAI locus); *UBE3A*, *SNRPN*, *NDN*, *SNURF* (SNURF-SNRPN locus); and *DNMT1*, *FDX1L* (DNMT1 locus).

### Gene expression simulation

The gene expression for a single coding gene is modeled using a set of coupled differential equations for the different processes and time-dependent numbers involved. Firstly, the count of active alleles of the DNA *n*(*t*) can be described by d*n*(*t*)/d*t* = *k*_on_ ⋅ (*N* − *n*(*t*)) − *k*_off_ ⋅ *n*(*t*), with the maximum number of active alleles *N*, which is 1 or 2 for monoallelically or biallelically expressed genes, respectively. Secondly, the number of mRNA molecules *m*(*t*) depends on the count of active alleles *n*(*t*) with d*m*(*t*)/d*t* = *k*_m_ ⋅ *n*(*t*) −* γ*_m_ ⋅ *m*(*t*) and thirdly, the number of protein molecules *p*(*t*) is modeled by d*p*(*t*)/d*t* = *k*_p_ ⋅ *m*(*t*) − *γ*_p_ ⋅ *p*(*t*), where the protein translation is proportional to the number of mRNA molecules *m*(*t*) (see Supplemental Methods).

### Data analysis

The genome alignment, counting, and ASE analysis of the mouse-brain data have been performed using GATK (4.4), SAMtools (1.10), subread (2.0), and STAR (2.7) with custom bash (5.0) and Python (3.11) scripts, running on Ubuntu (20.04). All statistical analyses and plots were done in Python (3.11) using the modules h5py (3.11), matplotlib (3.8), matplotlib-venn (0.11), numpy (2.0), pandas (2.2), and scipy (1.14). Gene ontology has been studied using Gorilla^44^.

### Statistics and reproducibility

The analysis uses already published scRNA-seq and scASE data. All data references are specified in the section of Data availability. The results may be reproduced by using the custom code described by in the section of Code availability.

### Reporting summary

Further information on research design is available in the Nature Portfolio Reporting Summary linked to this article.

## Supplementary information

Supplementary Information

Description of Additional Supplementary Materials

Supplementary Data 1

Supplementary Data 2

Supplementary Data 3

Supplementary Data 4

Supplementary Data 5

Supplementary Data 6

Supplementary Data 7

Supplementary Data 8

Supplementary Data 9

Supplementary Data 10

Supplementary Data 11

Reporting Summary

## Data Availability

Processed counts of scRNA-seq and scASE data are available from Zenodo: 3625024^11^, E-MTAB-9324 and E-MTAB-10714^13^, and GSE80810^10^. Co-expression clusters in human-iPSC data are available as Supplemental Data 5 in Cuomo et al.^11^. Imprinted gene annotations are available from Table S1 of Tucci et al.^1^ and from geneimprint (https://geneimprint.com). Additionally, we used data from Ensembl GRCh37 release 109 (https://grch37.ensembl.org) and GRCm38.p6 release 102 (https://nov2020.archive.ensembl.org). For the scASE exploration of the mouse-brain data, we aligned the scRNA-seq data available from SRP268938^12^ to the mouse genome GRCm38.p6 from (GENCODE release M25) using SNP information from the European Variation Archive (PRJEB43298).

## Code availability

The custom code used in this study for the re-analysis of scRNA-seq and scASE data is available at https://codeberg.org/epinoise/scbiasnoise (10.12751/g-node.xlvv9e). The custom code for the gene expression simulation is available at https://codeberg.org/epinoise/genexpsim (10.12751/g-node.89c3z3). The custom code for genome alignment and ASE counting of the mouse-brain data is available at https://codeberg.org/epinoise/star-align-ase. (10.12751/g-node.7knth1).
