# Supplementary material for: Monoallelic gene expression in developing cells increases genetic noise and Shannon entropy
Source: Commun Biol. 2025 Jun 4;8:857. doi: 10.1038/s42003-025-08128-2 (PMC12137721; doi:10.1038/s42003-025-08128-2)
Supplement: Supplementary file 1 — Supplementary Information [file 42003_2025_8128_MOESM1_ESM.pdf]

Supplemental Methods

Principal component analysis (PCA) and k-means clustering

We performed a PCA on all 36,044 single cells on the log normalised gene expression of 1,056 differentially expressed genes in the human iPSC data. These genes are the union of the 400 most different genes (estimated by one-way ANOVA test) for each of the comparisons of cells at day of differentiation 0 to 1, 1 to 2 and 2 to 3 and of 47 marker genes for iPSCs and endoderm (*CREB3L2*, *CXCR4*, *DNMT3B*, *DPPA2*, *DPPA4*, *ELF3*, *ELK3*, *EOMES*, *ETS2*, *ETV4*, *ETV5*, *FOXB1*, *FOXD3*, *FOXP1*, *GATA3*, *GATA4*, *GATA6*, *GSC*, *HHEX*, *HSP90B1*, *IRX3*, *KLF5*, *LEF1*, *LIN28A*, *MITF*, *MIXL1*, *MSX2*, *NANOG*, *NFE2L2*, *ONECUT1*, *PBX3*, *PLAGL1*, *PLSCR4*, *PODXL*, *POU5F1*, *PROM1*, *RARG*, *RFX2*, *SALL4*, *SOX17*, SOX2, *SP5*, *STAT1*, *T*, *TERT*, *ZBTB20* and *ZFP42*) (Cuomo et al.; Li et al.). The first four principal components (PCs) were then used to cluster the cells in four distinct clusters with k-means clustering. Each of the four clusters is matched to one day of differentiation. The matching efficiency ranges from 98.4% of the cells at day 0 matched to cluster A to 79.4% of cells at day 3 matched to cluster D. 3,765 cells, which could not be matched to the associated clusters, have been discarded in the analysis (***Figure 1—1***).

Measurement of observable quantities

To investigate the relation between genomic imprinting and genetic noise for different genes g, two observable quantities are needed: the genetic noise η and the bias of expression b for each gene g and cell population k. In the experiment each cell population k can be described by the following parameters: experimental dish e, day in vitro d, cell line l (from one human donor) and the number of cells $N(k)$.

Measurement of gene expression and genetic noise from scRNA-seq data

The scRNA-seq raw counts in counts per million (CPM) $x(g, j)$ for genes g and cells j are used to calculate the mean gene expression

$$\mu(g,k) \equiv \frac{1}{N(k)} \sum_{j\in k} x(g,j) \tag{1}$$

and the variance of gene expression

$$\sigma^2_\mu(g,k) \equiv \frac{1}{N(k)-1} \sum_{j\in k} (x(g,j) - \mu(g,k))^2, \tag{2}$$

calculated using Bessel's correction. The genetic noise is calculated by

$$\eta(g,k) \equiv \frac{\sigma_\mu(g,k)}{\mu(g,k)}. \tag{3}$$

The uncertainty on the genetic noise is not trivial to calculate and is extrapolated using a bootstrap method. It can be parameterized by

$$\sigma_\eta(g,k) \equiv \frac{\eta(g,k)}{\sqrt{2N(k)-1}}, \tag{4}$$

which shows a very good agreement with the estimate from simulation via bootstrap method (***Figure 1—10***). We also checked other measures of genetic noise, such as the distance to median, introduced by (Kar et al.). However, we found that the estimated CV^2 residual did not depend on the gene length, which might be because we used normalised counts (***Figure 1—11***). Further, we did not compare the genetic noise of different genes with each other. Instead, we investigated differences in genetic noise of each gene among cell populations.

Measurement of the genetic entropy and mutual information from gene expression distributions

We defined the genetic entropy for a gene g and a cell population k using the Kullback–Leibler divergence (relative entropy) by

$$h(g,k) \;=\; \sum_{x' \in [-5,\ldots,5]} p_{g,k}(x') \;\log\left(\frac{p_{g,k}(x')}{q_g(x')}\right), \tag{5}$$

where $p_{g,k}(x')$ and $q_g(x')$ are the probability density functions (PDFs) of gene expression for a gene g in a cell population k and all cells of all cell populations, respectively. For each gene g the gene expression $x(g,j)$ is log-normalized with $log(x+1)$ and linear-transformed such that the mean is 0 and the standard deviation is 1 to $x'(g,k)$. The PDFs are then estimated using the scaled gene expression for all cells (q_g) or for all cells in a cell population ($p_{g,k}$) by Gaussian kernel density estimation using Scott's bandwith estimation. Finally, the genetic entropy is calculated by the summing over the estimates at discrete points x' from −5.0 to +5.0 in distances of 0.1.

The mutual information between two genes g_1 and g_2 in a cell population k was estimated using the Pearson correlation coefficients $\rho(g_1;g_2,k)$ of log-normalized gene expression ($log(x+1)$) with

$$I(g_1;g_2,k) = -\frac{\log(1-\rho(g_1;g_2,k)^2)}{2}. \tag{6}$$

Measurement of the expression bias from scASE data

The estimation of the expression bias is based on the scASE counts for the SNPs i and cells j. The counts for the reference and the alternative allele are denoted as $r(i,j)$ and $a(i,j)$, respectively. As the cells come from different human donors, the SNPs are different for different cell lines and for each SNP it is unknown whether the reference or the alternative allele is at the paternal or maternal allele. An expression bias sign factor for each SNP i and cell line l is defined based on the contained cells j as

$$\beta(i,l) \equiv \frac{\sum_{j\in l} r(i,j) - a(i,j)}{\left|\sum_{j\in l} r(i,j) - a(i,j)\right|}. \tag{7}$$

Then the expression bias per SNP i and cell j can be defined as

$$b_{ij}(i,j) \equiv \beta(i,l(j)) \cdot \frac{r(i,j)-a(i,j)}{r(i,j)+a(i,j)}, \tag{8}$$

which is +1 or −1 for monoallelic expression of the most commonly expressed or the other allele, respectively, and ≈ 0 in the case of biallelic expression.

The expression bias per gene g and cell j is then defined as weighted average over SNPs i associated to gene g using the total counts $t(i,j) = r(i,j) + a(i,j)$ by

$$b_j(g,j) \equiv \frac{1}{\sum_{i\in g} t(i,j)} \sum_{i\in g} t(i,j) \cdot b_{ij}(i,j), \tag{9}$$

which is ≈ 0 for biallelic and +1 for monoallelic expression of the most common allele. A value of −1 corresponds to monoallelic expression of the opposite allele. The variance of this value is estimated by

$$\sigma^2_{b_j}(g,j) \equiv \frac{1}{\sum_{i\in g} t(i,j)}. \tag{10}$$

The average expression bias per gene g and cell population k we defined by

$$\langle b\rangle(g,k) \equiv \frac{1}{t_b(g,k)} \sum_{i\in g} \sum_{j\in k} t(i,j) \cdot b_j(g,j) \tag{11}$$

with its variance

$$\sigma^2_b(g,k) \equiv \frac{1}{t_b(g,k)}, \tag{12}$$

where the total count used for the expression bias calculation is

$$t_b(g,k) \equiv \sum_{i\in g}\sum_{j\in k} t(i,j). \tag{13}$$

With this definition the average expression bias may be negative. As we were not interested in the parent of origin, the expression bias is defined as the absolute of the average expression bias by

$$b(g,k) \equiv |\langle b\rangle(g,k)|\,, \tag{14}$$

which is a value between 0 for fully biallelic and 1 for monoallelic expression. In the section where we investigate the impact of the parent of origin we instead use the estimate of the average expression bias which ranges from −1 to +1.

Quality assessment of cell populations

To achieve a robust statistical analysis of a variable, e.g. the genetic noise $\eta(g_2,k)$ of a gene g_2, which is dependent on the expression bias $b(g_1,k)$ of a gene g_1 some quality assessment is performed.

The gene expression of the investigated gene g_2 for the dependent variable must satisfy

$$\mu(g_2,k) \geq 0.2 \text{ CPM}. \tag{15}$$

For all data points of cell populations and genes with scRNA-seq data available 97.3% pass this selection (***Figure 1—2A,B***).

For a gene g_1 the cell populations k must have the bias information available for at least 5 cells j,

$$N_b(g_1,k) \geq 5. \tag{16}$$

Further, the bias calculation must be done with a total count of at least 25,

$$t_b(g_1,k) \geq 25, \tag{17}$$

equivalent to a maximum estimated expression bias uncertainty of 0.2,

$$\sigma_b(g_1,k) = \frac{1}{\sqrt{t_b(g_1,k)}} \leq 0.2. \tag{18}$$

Out of 59,455 data points of 517 cell populations and 115 imprinted genes with scASE data available 35,419 have at least one cell with bias information available and 22,839 pass these selection criteria (***Figure 1—3***).

Statistical analysis

To assess the quantitative change of a dependent variable $y(g_2,k)$ from the expression bias $b(g_1,k)$ of a gene g_1 and the day of differentiation $d(k)$ we perform a weighted multiple linear regression with the inverse variances of the dependent variable $1/\sigma_y^2(g_2,k)$ as weights. The dependent variable is here either the genetic noise $\eta(g_2,k)$ or the gene expression $\mu(g_2,k)$ of a gene g_2. The criterion to be minimized is

$$Q \equiv \sum_k \frac{(y(g_2,k) - \phi(g_1,k,\hat{\mathbf{p}}))^2}{\sigma_y^2(g_2,k)} \tag{19}$$

with a function $\phi(g_1,k,\hat{\mathbf{p}})$ depending on the cell population k and the estimated parameters vector $\hat{\mathbf{p}}$. As the dependent variable $y(g_2,k)$ may not only depend on the expression bias $b(g_1,k)$ but also on the day of differentiation $d(k)$, the dependent variable can be parameterized as a function of b and d as

$$f(b,d,\hat{\mathbf{p}}) \equiv f(b,d,(\hat{y}_0,\hat{\xi}_b^y,\hat{\xi}_d^y)) \equiv \hat{y}_0 \cdot \left(\left(\hat{\xi}_b^y - 1\right)\cdot b + 1\right) \cdot \left(\frac{\hat{\xi}_d^y - 1}{3}\cdot d + 1\right). \tag{20}$$

By this choice all estimated parameters have a dedicated meaning. $\hat{y}_0$ is the estimated value of the dependent variable at expression bias $b = 0$ (biallelic expression) and day of differentiation $d = 0$ as

$$f(b=0,d=0,\hat{\mathbf{p}}) = \hat{y}_0, \tag{21}$$

$\hat{\xi}^y_b$ is the estimated change of the dependent variable from bi- to monoallelic expression as

$$\frac{f(b=1,d,\hat{\mathbf{p}})}{f(b=0,d,\hat{\mathbf{p}})} = \hat{\xi}^y_b \tag{22}$$

and $\hat{\xi}^y_d$ is the estimated change of the dependent variable from day of differentiation 0 to 3 as

$$\frac{f(b,d=3,\hat{\mathbf{p}})}{f(b,d=0,\hat{\mathbf{p}})} = \hat{\xi}^y_d. \tag{23}$$

A value of $\hat{\xi}^y_{b/d} > 1$ corresponds to an increase and $\hat{\xi}^y_{b/d} < 1$ to a decrease of the dependent variable. In the case of $\hat{\xi}^y_b = 1$ or $\hat{\xi}^y_d = 1$, the dependent variable is not dependent on b or d, respectively. The function definition depends on the assumption that the dependent variable has a linear dependency both on the bias of expression b and the day of differentiation d independently. With the definition in ***Equation 20*** the function to be put in ***Equation 19*** can be written as

$$\phi(g_1,k,\hat{\mathbf{p}}) \equiv f(b(g_1,k),d(k),\hat{\mathbf{p}}). \tag{24}$$

Two examples of genetic noise in dependency of both expression bias and day of differentiation shown in ***Figure 2—4***. An alternative linear regression has been checked, where the dependent variable is only dependent on the expression bias b and not on the day of differentiation d by fixing $\hat{\xi}^y_d = 1$. For the 108 imprinted genes with at least 10 cell populations the regression with free and fixed $\hat{\xi}^y_d$ result in compatible values of genetic noise change from bi- to monoallelic expression $\hat{\xi}^y_b$ (***Figure 2—5A***). Furthermore, both the estimated uncertainty on $\hat{\xi}^y_b$ and the residual variance are smaller or approximately the same when going from regression with fixed to free $\hat{\xi}^y_d$ (***Figure 2—5A,B***). It also has been checked that the estimates of genetic noise change from bi- to monoallelic expression and from day 0 to day 3 are not highly correlated (***Figure 2—5C,D***).

In the study of the parent of origin we performed a linear regression with 4 parameters, splitting ξ^η_b into genetic noise change from bi- to monoallelic expression ξ^η_{b+} and from bi- to inversely monoallelic expression ξ^η_{b-}, replacing in ***Equation 20***:

$$\left(\hat{\xi}^\eta_b - 1\right)\cdot b \ \text{ by } \begin{cases} \left(\hat{\xi}^\eta_{b-} - 1\right)\cdot\langle b\rangle, & \text{if } \langle b\rangle < 0 \\ \left(\hat{\xi}^\eta_{b+} - 1\right)\cdot\langle b\rangle, & \text{if } \langle b\rangle \geq 0 \end{cases} \tag{25}$$

and with the average expression bias $\langle b\rangle$ instead of its absolute b. For many genes, the regression quality suffers from low numbers of cell populations with negative average expression bias (***Figure 1—3C***).

Similarly, for studying the impact of the sex of the donor we split ξ^η_b into the genetic noise change for cell populations from female donors ($\xi^\eta_{b,\mathrm{f}}$) and from male donors ($\xi^\eta_{b,\mathrm{m}}$).

The p-value of the change testing the null-hypothesis of $\xi^y_{b/d} = 1$ is approximated using the cumulative distribution function of the student's t continuous random variable:

$$p\left(\hat{\xi}^y_{b/d} \neq 1\right) \approx \mathrm{cdf}_t\left(\frac{\left|\hat{\xi}^y_{b/d} - 1\right|}{\hat{\sigma}(\xi^y_{b/d})}, N_{\mathrm{dof}}\right) \tag{26}$$

with the estimated uncertainty on the change $\hat{\sigma}(\xi^y_{b/d})$ and the number of degrees of freedom $N_{\mathrm{dof}} = N_k - N_p$, where N_k is the number of cell populations considered and $N_p = 3$ is the number of parameters used in the linear regression.

We estimated the contributions of extrinsic (due to differentiation) and intrinsic (due to differential gene expression) sources to genetic noise change from day 0 to day 3 of differentiation using a linear regression with the function

$$\xi^\mu_b(\xi^\eta_b,(\hat{\kappa},\hat{\beta})) = \exp\left(-\hat{\kappa}\cdot\log\left(\frac{\xi^\eta_b}{\hat{\beta}}\right)\right). \tag{27}$$

The extrinsic genetic noise change is estimated using the parameter $\hat{\kappa}$ with

$$\xi^{\eta}_{d,\mathrm{diff}} = \xi^{\eta}_{d} \exp\left(\frac{\log\left(\xi^{\mu}_{d}\right)}{\hat{\kappa}}\right) \tag{28}$$

and the intrinsic genetic noise change is then

$$\xi^{\eta}_{d,\mathrm{expr}} = \frac{\xi^{\eta}_{d}}{\xi^{\eta}_{d,\mathrm{diff}}} = \exp\left(\frac{-\log\left(\xi^{\mu}_{d}\right)}{\hat{\kappa}}\right). \tag{29}$$

Simulation of gene expression in a gene regulatory network with permissive and repressive gene-gene interaction

The gene expression for a single coding gene is modeled using a set of coupled differential equations for the different processes and time dependent numbers involved. Firstly, the count of active alleles of the DNA $n(t)$ can be described by

$$\frac{\mathrm{d}}{\mathrm{d}t} n(t) = \underbrace{k_{\mathrm{on}} \cdot (\tilde{n} - n(t))}_{\text{DNA activation}} - \underbrace{k_{\mathrm{off}} \cdot n(t)}_{\text{DNA inactivation}} \tag{30}$$

with the maximum number of active alleles $\tilde{n}$, which is 1 or 2 for monoallelically or biallelically expressed genes, respectively. Secondly, the number of mRNA molecules $m(t)$ depends on the count of active alleles $n(t)$ with

$$\frac{\mathrm{d}}{\mathrm{d}t} m(t) = \underbrace{k_{\mathrm{m}} \cdot n(t)}_{\text{mRNA transcription}} - \underbrace{\gamma_{\mathrm{m}} \cdot m(t)}_{\text{mRNA degradation}} \tag{31}$$

and lastly, the number of protein molecules $p(t)$ is modeled by

$$\frac{\mathrm{d}}{\mathrm{d}t} p(t) = \underbrace{k_{\mathrm{p}} \cdot m(t)}_{\text{protein translation}} - \underbrace{\gamma_{\mathrm{p}} \cdot p(t)}_{\text{protein degradation}} , \tag{32}$$

where the protein translation is proportional to the number of mRNA molecules $m(t)$ (***Figure 2—12A***).

A simplified gene regulatory network is simulated with one expressed and one repressed gene (***Figure 2—12B***). The expression of the expressed gene is modulated by the protein molecules of the imprinted gene, which act as a transcription factor to the DNA of the expressed gene. Its count of active alleles $n^{\mathrm{e}}(t)$ therefore depends on the number of proteins of the imprinted gene $p^{\mathrm{i}}(t)$. The time dependency can be written as modified version of ***Equation 30*** with

$$\frac{\mathrm{d}}{\mathrm{d}t} n^{\mathrm{e}}(t) = \underbrace{k^{\mathrm{i}\to\mathrm{e}}_{\mathrm{on}} \cdot p^{\mathrm{i}}(t) \cdot (\tilde{n}^{\mathrm{e}} - n^{\mathrm{e}}(t))}_{\text{modulated DNA activation}} - \underbrace{k^{\mathrm{e}}_{\mathrm{off}} \cdot n^{\mathrm{e}}(t)}_{\text{DNA inactivation}} , \tag{33}$$

where $k^{\mathrm{i}\to\mathrm{e}}_{\mathrm{on}}$ describes the strength of permissive gene-gene interaction. The expression of the repressed gene is simulated for a process where the proteins of the imprinted gene inactivate the DNA of the repressed gene. The count of active alleles of the repressed gene $n^{\mathrm{r}}(t)$ is therefore dependent on the number of proteins of the imprinted gene $p^{\mathrm{i}}(t)$ via modified ***Equation 30*** with

$$\frac{\mathrm{d}}{\mathrm{d}t} n^{\mathrm{r}}(t) = \underbrace{k^{\mathrm{r}}_{\mathrm{on}} \cdot (\tilde{n}^{\mathrm{r}} - n^{\mathrm{r}}(t))}_{\text{DNA activation}} - \underbrace{k^{\mathrm{i}\dashv\mathrm{r}}_{\mathrm{off}} \cdot p^{\mathrm{i}}(t) \cdot n^{\mathrm{r}}(t)}_{\text{modulated DNA inactivation}} , \tag{34}$$

where $k^{\mathrm{i}\dashv\mathrm{r}}_{\mathrm{off}}$ describes the strength of repression.

The choice of the nominal constants of gene expression, given in ***Figure 2—13A***, is motivated by previous measurements of the mouse gene *Prl2C2* rates (Suter et al.).

In the case of monoallelic expression of the imprinted gene ($\tilde{n}^{\mathrm{i}} = 1$), the dosage is compensated by up-regulation of the mRNA transcription rate k_{m} by a factor of two. We performed multiple

simulations modifying the 6 process rates of the imprinted gene from 0.1 to 10 times the nominal value (0.1, 0.2, 0.5, 1, 2, 5 and 10 times, ***Figure 2—13***).

We simulated events for the different processes and genes at discrete time points t_j. Event $j+1$ was simulated using the calculated rates $r_i(t_j)$ for the different processes i after event j happened. The total absolute rate is $r(t_j) \equiv \sum_i \left|r_i(t_j)\right|$. Each new event was simulated by two random numbers: one for the time point $t_{j+1} = t_j + \Delta t_j$ with Δt_j being randomly distributed with an exponential probability density function $\sim r(t_j)e^{-r(t_j)\Delta t_j}$ and one for the chosen process $i(t_j)$ with process probabilities $P_i(t_j) = \frac{r_i(t_j)}{r(t_j)}$.

References

[Cuomo et al.] Cuomo, A. S. E., Seaton, D. D., McCarthy, D. J., Martinez, I., Bonder, M. J., Garcia-Bernardo, J., Amatya, S., Madrigal, P., Isaacson, A., Buettner, F., Knights, A., Natarajan, K. N., Vallier, L., Marioni, J. C., Chhatriwala, M., and Stegle, O. Single-cell RNA-sequencing of differentiating iPS cells reveals dynamic genetic effects on gene expression. 11(1):810.

[Kar et al.] Kar, G., Kim, J. K., Kolodziejczyk, A. A., Natarajan, K. N., Torlai Triglia, E., Mifsud, B., Elderkin, S., Marioni, J. C., Pombo, A., and Teichmann, S. A. Flipping between Polycomb repressed and active transcriptional states introduces noise in gene expression. 8(1):36.

[Li et al.] Li, L.-C., Wang, X., Xu, Z.-R., Wang, Y.-C., Feng, Y., Yang, L., Qiu, W.-L., Yang, L., Yu, X.-X., Gu, J., and Xu, C.-R. Single-cell patterning and axis characterization in the murine and human definitive endoderm. 31(3):326–344.

[Suter et al.] Suter, D. M., Molina, N., Gatfield, D., Schneider, K., Schibler, U., and Naef, F. Mammalian genes are transcribed with widely different bursting kinetics. 332(6028):472–474.

Supplemental Figures

A

B

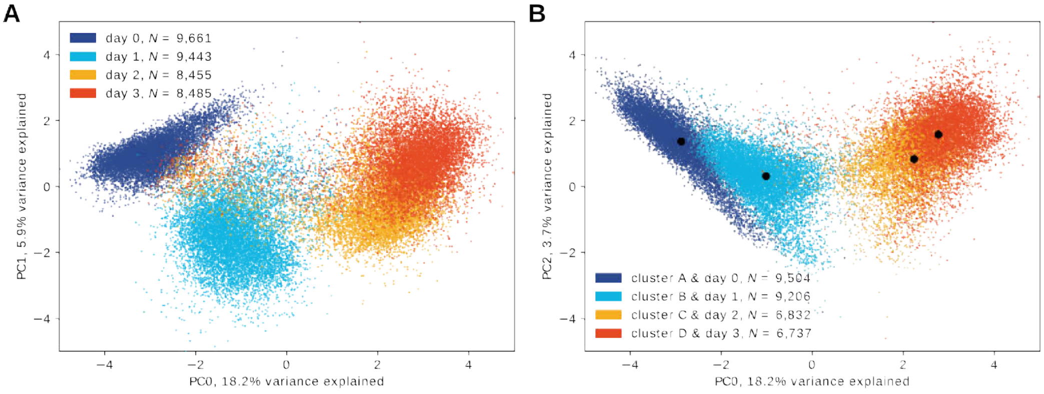

C

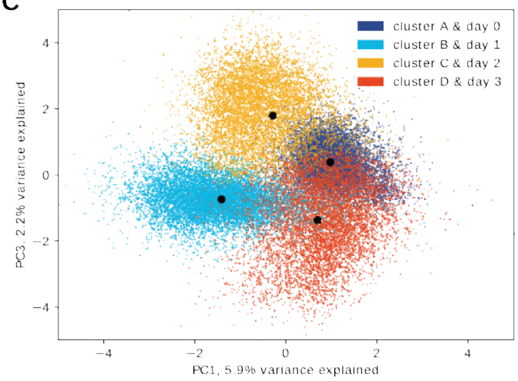

D

cluster	day 0	day 1	day 2	day 3
A	9,504	142	465	342
B	12	9,206	480	59
C	5	9	6,832	1,347
D	140	86	678	6,737
all	9,661	9,443	8,455	8,485

Figure S1—1. Principal component analysis and k-means clustering of gene expression in human-iPSC data. Related to ***Figure 1A–E***. (**A**) PCA on 1,056 genes for 36,044 cells. The distribution of the first two principal components is shown, colored by the day of differentiation. (**B–C**) Distribution of principal components 0 and 2 (**B**) and 1 and 3 (**C**) after k-means clustering with 4 clusters and matching the resulting clusters A–D to the days of differentiation 0–3. Only the cells after the matching are shown and the clusters show good separation in the 4-dimensional space of the first four principal components. (**D**), Number of cells in the clusters A–D which are at days of differentiation 0–4. In the off-diagonal elements there are the number of cells which we discarded in the analysis (in total 3,765). In particular, the cells of days 1–3 in cluster A may be classified as cells with failed differentiation (25% of discarded cells) and cells in cluster B at days 2–3 and cells in cluster C at day 3 as cells with delayed differentiation (75% of discarded cells).

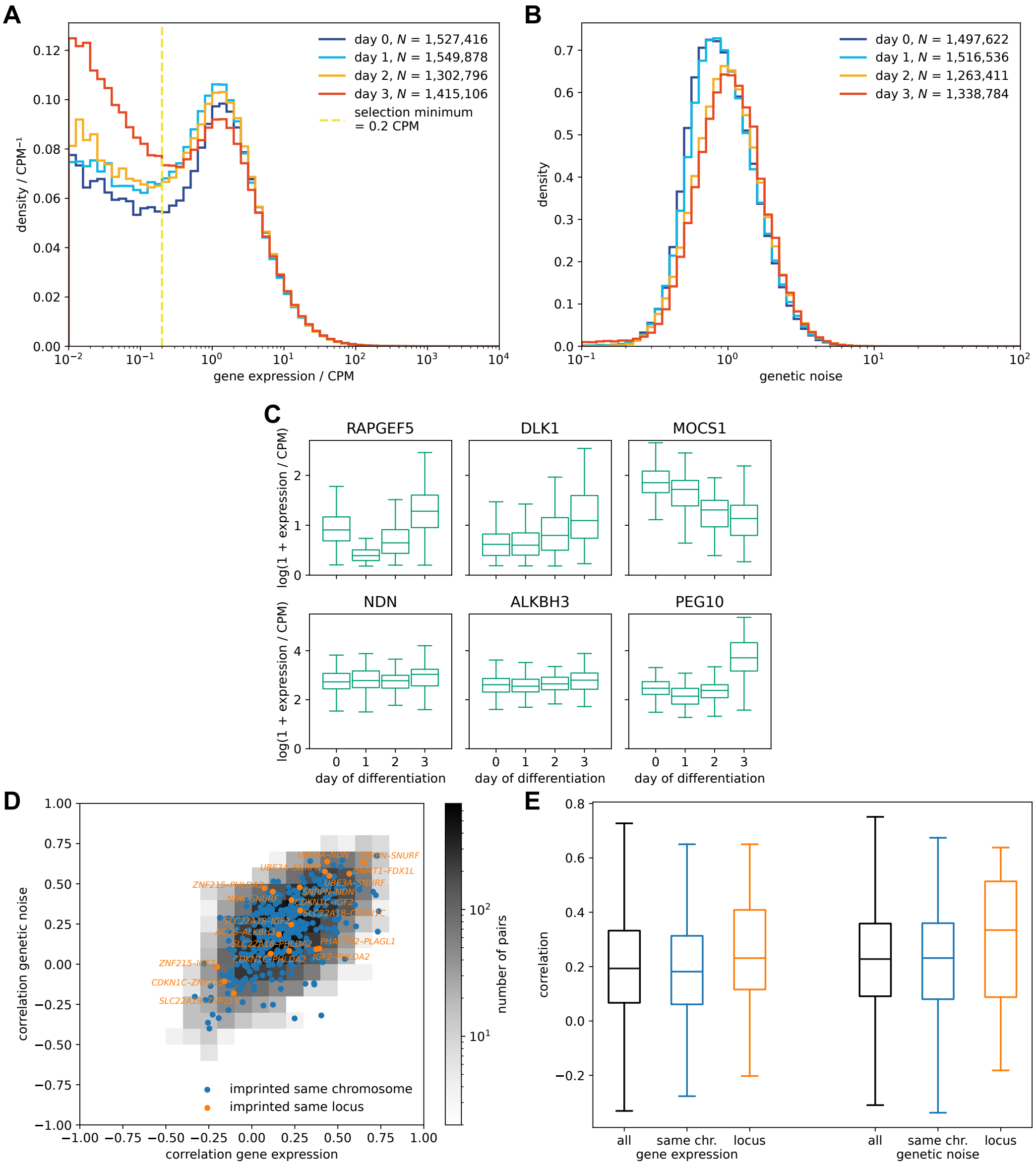

Figure S1—2. Distributions of observable quantities from human-iPSC scRNA-seq data for different days of differentiation. Each entry in the histograms corresponds to one cell population k and one gene g out of all genes with scRNA-seq data available. Related to ***Figure 1A–E***. (**A**) Gene expression. (**B**) Genetic noise after applying the minimum gene expression selection of $\mu(g, k) \geq 0.2$ CPM. (**C**) Gene expression of some imprinted genes in dependency of the day of differentiation. $N = 415$, 404, 501, 513, 504 and 516 cell populations for *RAPGEF5*, *DLK1*, *MOCS1*, *NDN*, *ALKBH3* and *PEG10*, respectively. (**D**) Linear dependency of gene expression and genetic noise correlation. Distribution for all imprinted gene pairs, points for imprinted gene pairs on same chromosome and imprinted gene pairs from same locus. (**E**) Boxplots of gene expression and genetic noise correlation for imprinted gene pairs. (**D,E**) N = 13,110 pairs of imprinted genes, N = 396 pairs of imprinted genes on same chromosome, and N = 19 pairs of imprinted genes on same locus.

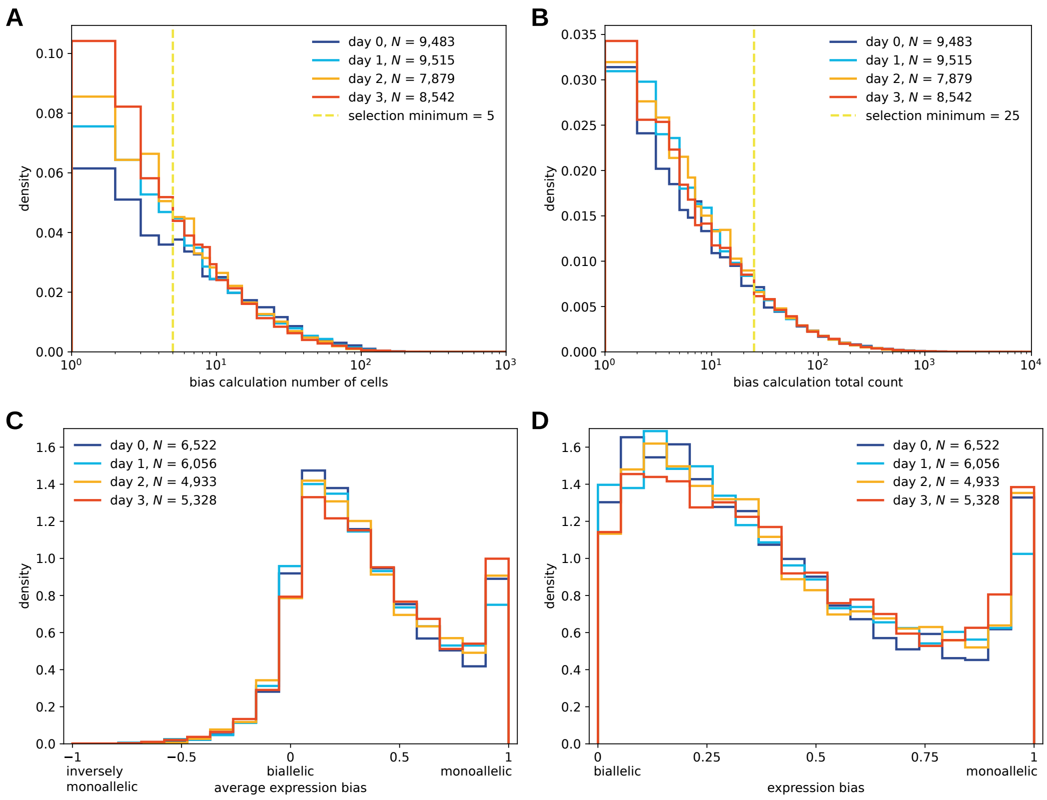

Figure S1—3. Distributions of observable quantities from human-iPSC scASE data for different days of differentiation. Each entry in the histograms corresponds to one cell population k and one gene g out of all imprinted genes with scASE data available. Related to ***Figure 1F–I***. (**A**) Number of cells $N_b(g,k)$ used for the cell population's expression bias calculation. (**B**) Total count $t_b(g,k)$ used for the cell population's expression bias calculation. (**C**) Average expression bias after applying the selection cuts of $N_b(g,k) \geq 5$ and $t_b(g,k) \geq 25$. (**D**) Expression bias (absolute of average expression bias) after applying the selection cuts of $N_b(g,k) \geq 5$ and $t_b(g,k) \geq 25$.

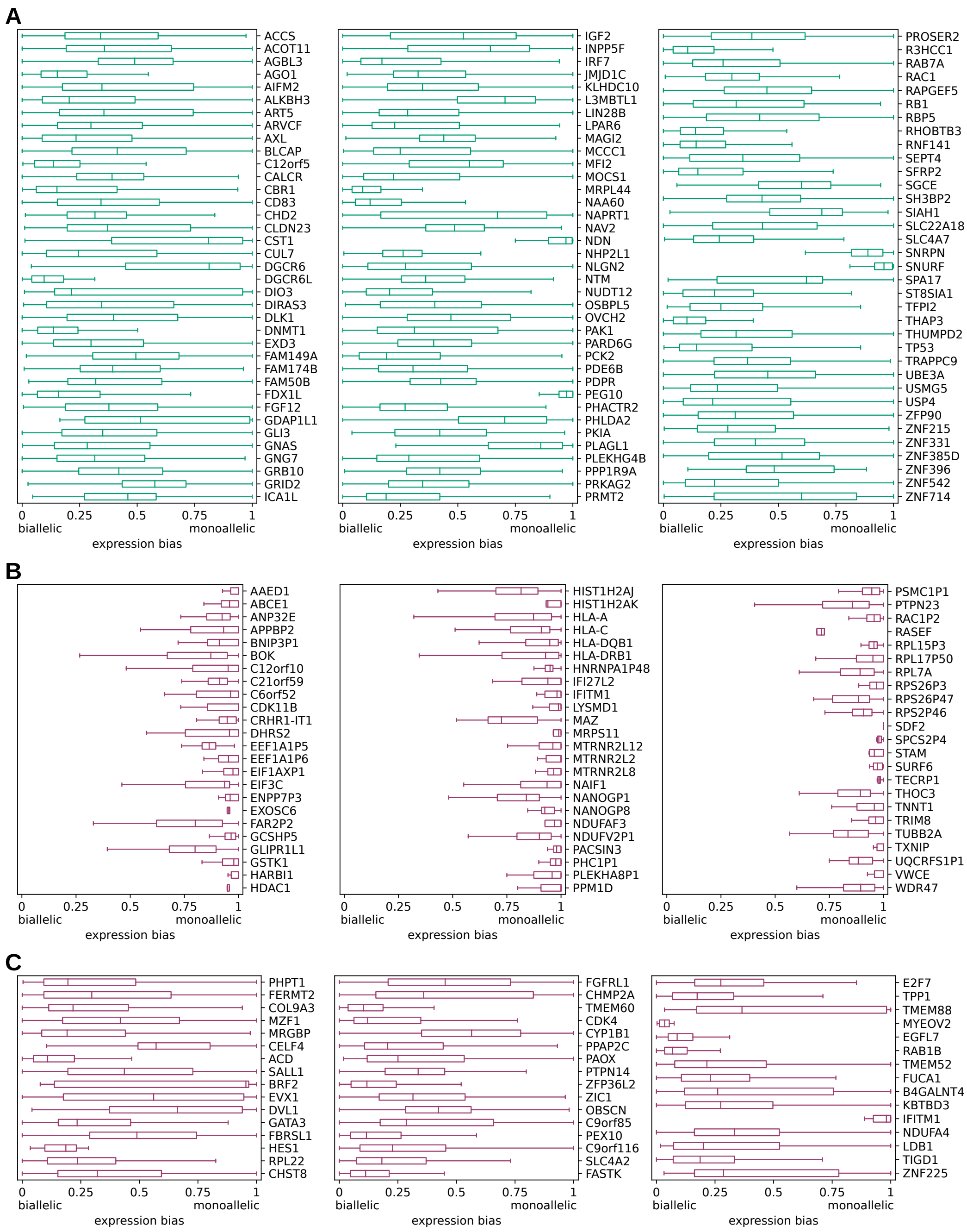

Figure S1—4. Expression bias of imprinted genes, non-imprinted genes and imprinted gene candidates in human-iPSC data. Related to ***Figure 1I***. (**A**) 109 imprinted genes have at least 5 cell populations after quality assessment for bias calculation (Methods). Of these, the 4 genes *NDN*, *PEG10*, *SNRPN* and *SNURF* are rather monoallelically expressed. (**B**) For 71 of 10,048 non-imprinted genes, which have at least 5 cell populations after quality assessment for bias calculation, the cell populations have an expression bias of $b > 1/3$ with $p < 0.05$ (approximated by one-way ANOVA test). *IFITM1* has been previously reported as an imprinted gene candidate. (**C**) 47 genes, which are expressed in the data, are predicted to be imprinted. Of these, only *IFITM1* is clearly monoallelically expressed.

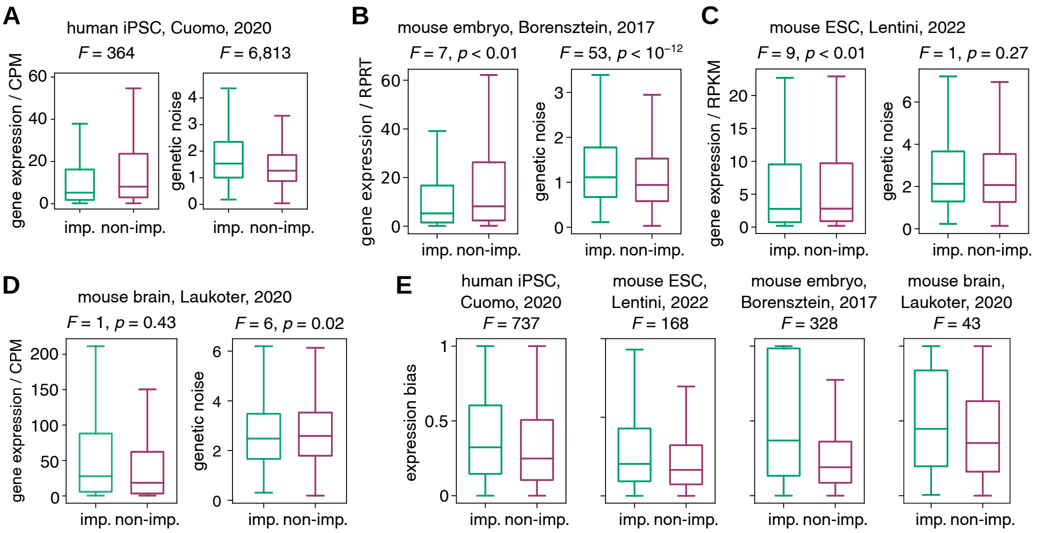

Figure S1—5. Gene expression and genetic noise (**A–D**) and expression bias (**E**) of imprinted and non-imprinted genes in different datasets. F-statistics and p-values are estimated using one-way ANOVA-test. Related to ***Figure 1***. (**A**) N = 55,048 and 5,480,952 cell populations for imprinted and non-imprinted genes, respectively. (**B**) N = 1,141 and 124,029 cell populations for imprinted and non-imprinted genes, respectively. (**C**) N = 4,320 and 467,602 cell populations for imprinted and non-imprinted genes, respectively. (**D**) N = 1,898 and 230,546 cell populations for imprinted and non-imprinted genes, respectively. (**E**) N = 22,839 and 2,026,759 cell populations in human iPSC differentiation for imprinted and non-imprinted genes, respectively. N = 2,274 and 246,120 cell populations in mouse ESC differentiation for imprinted and non-imprinted genes, respectively. N = 304 and 43,228 cell populations in mouse embryonic development for imprinted and non-imprinted genes, respectively. N = 489 and 51,365 cell populations in mouse brain development for imprinted and non-imprinted genes, respectively.

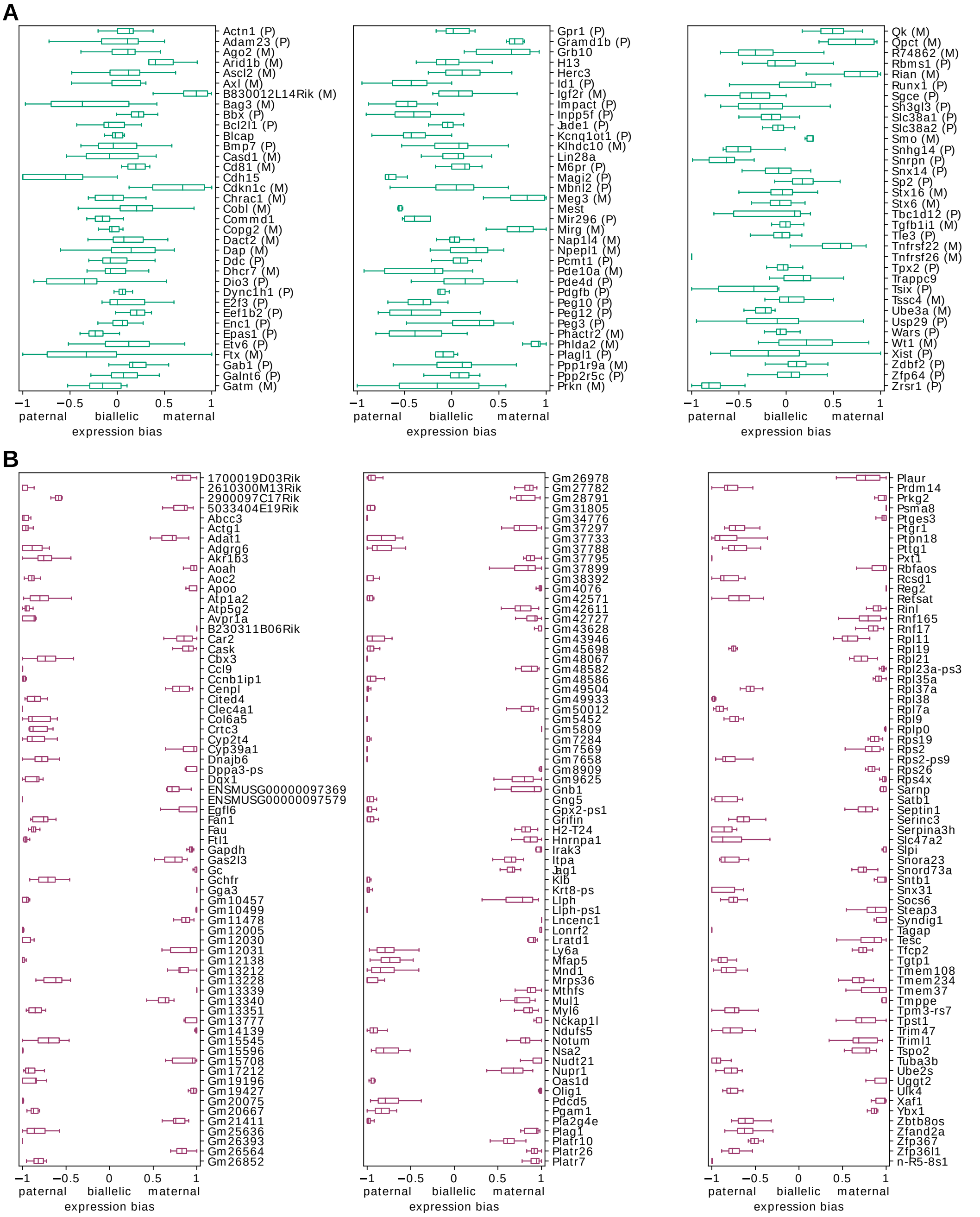

Figure S1—6. Expression bias of imprinted and non-imprinted genes in mouse-ESC data. Related to ***Figure 1I***. (**A**) 104 imprinted genes have at least 5 cell populations after quality assessment for bias calculation (Methods). Of these, the 4 genes *Gramd1b*, *Meg3*, *Phlda2* and *Rian* are rather maternally and the 2 genes *Tnfrsf26* and *Zrsr1* are rather paternally expressed. For *Gramd1b* and *Tnfrsf26* opposite paternal expression is reported in literature, In brackets the reported maternal (M) or paternal (P) expression is given. (**B**) For 207 of 10,440 non-imprinted genes, which have at least 5 cell populations after quality assessment for bias calculation, the cell populations have an expression bias of $b > 1/3$ or $b < -1/3$ with $p < 0.05$ (approximated by one-way ANOVA test).

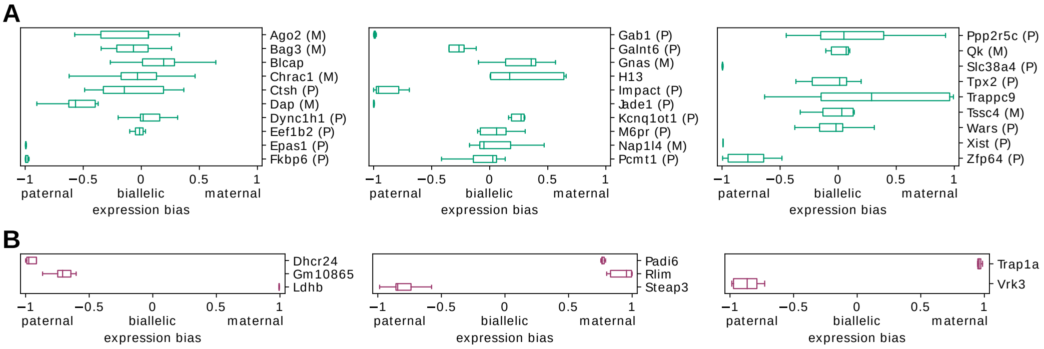

Figure S1—7. Expression bias of imprinted and non-imprinted genes in mouse-embryo data. Related to ***Figure 1I***. (**A**) 29 imprinted genes have at least 5 cell populations after quality assessment for bias calculation (Methods). Of these, the 7 genes *Epas1*, *Fkbp6*, *Gab1*, *Impact*, *Jade1*, *Slc38a4* and *Xist* are rather paternally expressed, as reported in literature. In brackets the reported maternal (M) or paternal (P) expression is given. (**B**) For 8 of 4,296 non-imprinted genes, which have at least 5 cell populations after quality assessment for bias calculation, the cell populations have an expression bias of $b > 1/3$ or $b < -1/3$ with $p < 0.05$ (approximated by one-way ANOVA test).

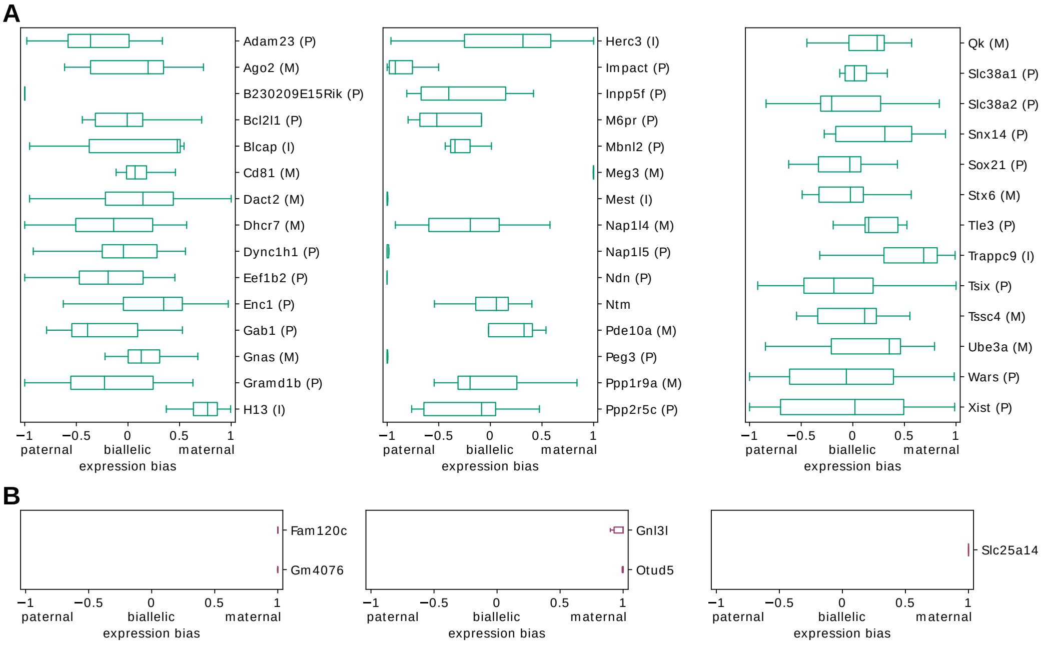

Figure S1—8. Expression bias of imprinted and non-imprinted genes in mouse-brain data. Related to ***Figure 1I***. (**A**) 43 imprinted genes have at least 5 cell populations after quality assessment for bias calculation (Methods). Of these, the 4 genes *B230209E15Rik*, *Nap1l5*, *Ndn* and *Peg3* are rather paternally expressed, as reported in literature. The in literature described maternally expressed *Meg3* is found maternally expressed. The gene *Mest* is found rather paternally expressed. In brackets the reported maternal (M) or paternal (P) expression is given. (**B**) For 5 of 4,508 non-imprinted genes, which have at least 5 cell populations after quality assessment for bias calculation, the cell populations have an expression bias of $b > 1/3$ or $b < -1/3$ with $p < 0.05$ (approximated by one-way ANOVA test).

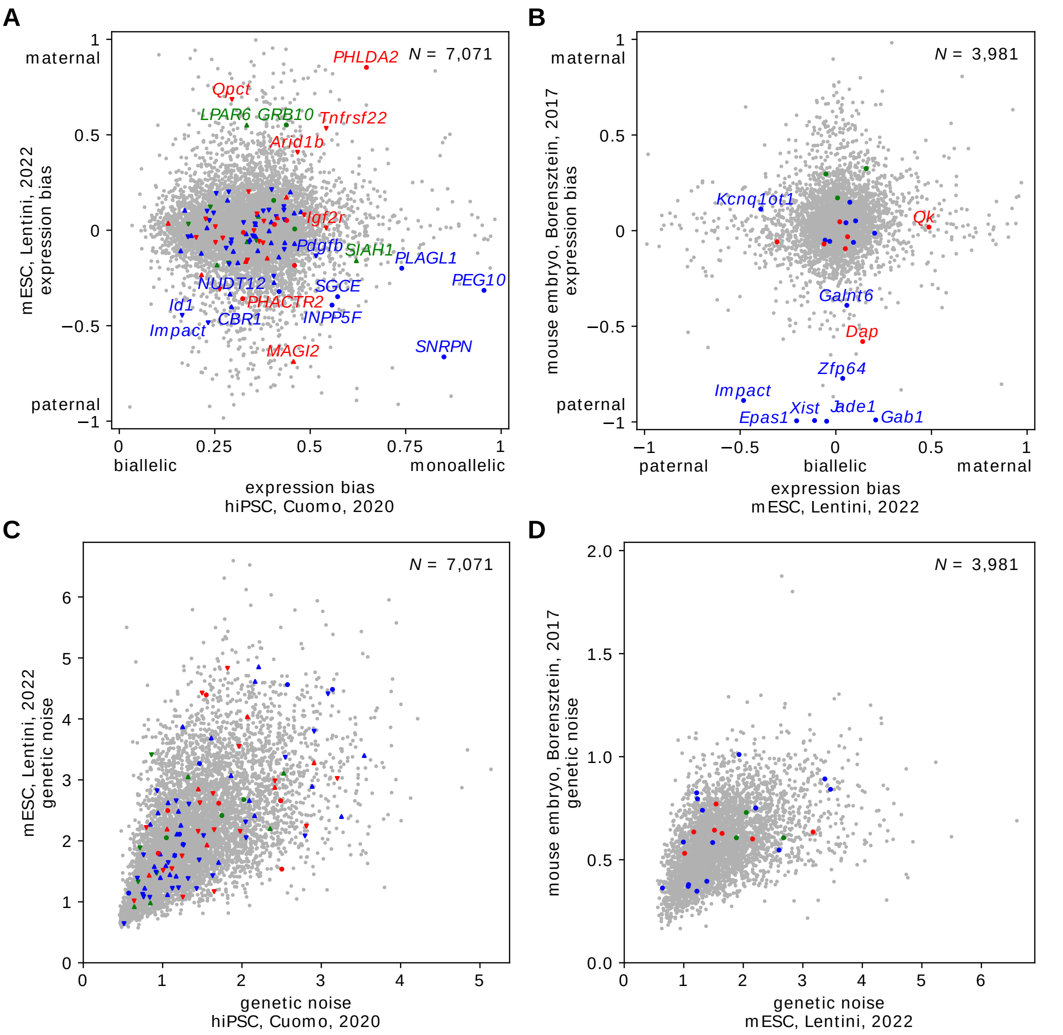

Figure S1—9. Correlation of expression bias and genetic noise between different datasets. Related to ***Figure 1***. Colored points indicate imprinted genes (red: maternally expressed, blue: paternally expressed, green: unknown). (**A**) Mean expression bias of orthologous genes of human iPSC and mouse ESC data. Genes which are imprinted both in human and mouse are marked in boldface. (**B**) Mean expression bias of mouse genes found both in mouse ESC data and mouse embryon data. (**C**) Mean genetic noise for orthologous genes of human iPSC and mouse ESC data. (**D**) Mean genetic noise for mouse genes found both in mouse ESC data and mouse embryo data.

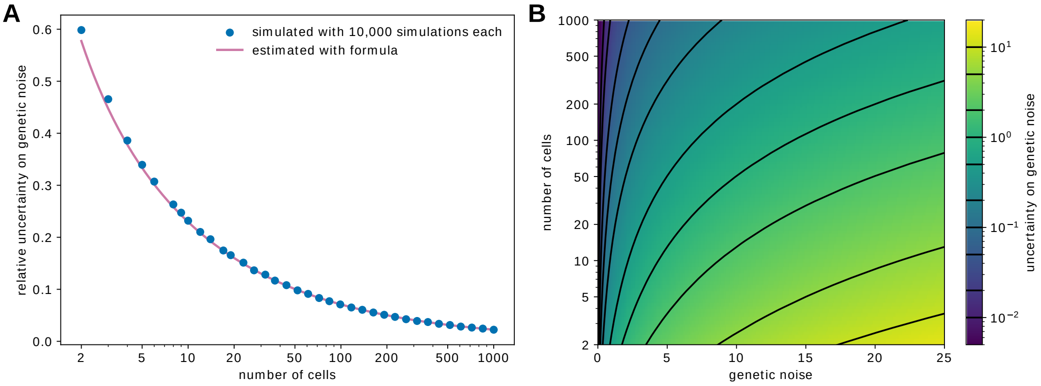

Figure S1—10. Estimate of the uncertainty on genetic noise. Related to ***Figure 1A–E***. (**A**) The relative uncertainty on genetic noise in dependency of the number of cells. The estimate with formula (line) compares well with the simulation via bootstrap method with 10,000 simulations for each number of cells. (**B**) The calculated estimate of the uncertainty on genetic noise in dependency of the genetic noise and number of cells.

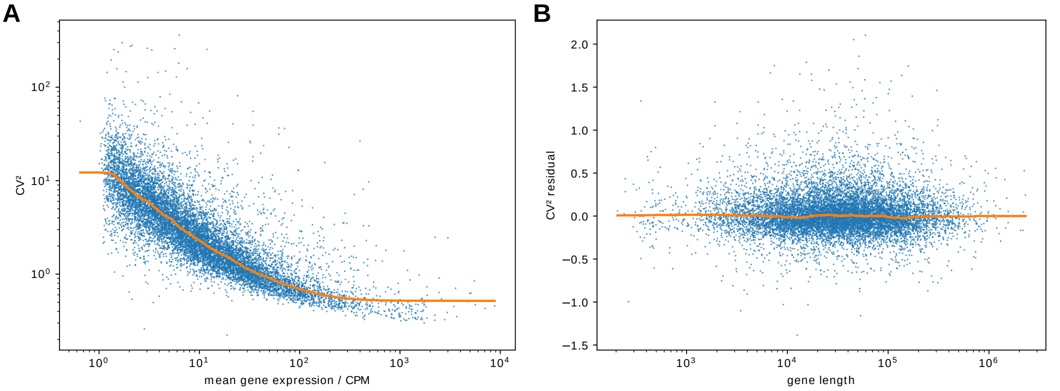

Figure S1—11. Dependency of genetic noise on the gene expression and gene length in human-iPSC data. Reletad to ***Figure 1A–E***. (**A**) The squared coefficient of variation (CV^2) in dependency on the mean gene expression for all 11,231 genes. The solid line shows the median-filtered estimate. (**B**) The CV^2 residual, calculated as difference of CV^2 and its median filtered estimate, in dependency on the gene length. Again, the solid line shows the median-filtered estimate.

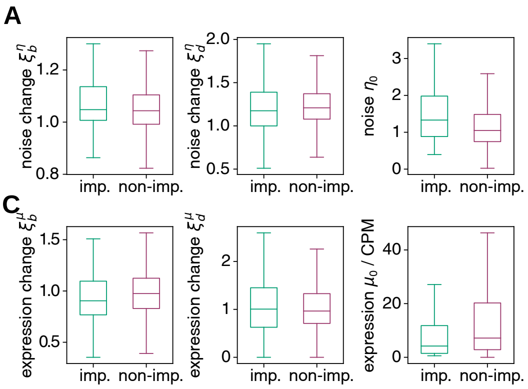

B

	imprinted	non-imprinted	F	p
number of genes N	108	9,900		
noise change ξ_b^η	$1.05^{+0.09}_{-0.04}$	$1.04^{+0.06}_{-0.05}$	0.21	0.65
noise change ξ_d^η	$1.17^{+0.21}_{-0.18}$	$1.21^{+0.16}_{-0.13}$	0.13	0.71
noise η_0	$1.33^{+0.65}_{-0.45}$	$1.05^{+0.44}_{-0.30}$	35.4	$< 10^{-8}$

D

	imprinted	non-imprinted	F	p
number of genes N	104	9,829		
expression change ξ_b^μ	$0.90^{+0.19}_{-0.14}$	$0.97^{+0.15}_{-0.15}$	0.01	0.91
expression change ξ_d^μ	$1.01^{+0.45}_{-0.38}$	$0.97^{+0.36}_{-0.26}$	0.02	0.88
expression μ_0 / CPM	$4.20^{+7.61}_{-2.73}$	$7.16^{+13.1}_{-4.33}$	0.35	0.56

E

	imprinted	non-imprinted	F	p
number of genes N'	102	9,687		
noise change ξ_d^η	$1.18^{+0.21}_{-0.13}$	$1.21^{+0.16}_{-0.13}$	0.06	0.81
noise change $\xi_{d,\mathrm{diff}}^\eta$	$1.20^{+0.08}_{-0.10}$	$1.20^{+0.07}_{-0.06}$	0.13	0.72
noise change $\xi_{d,\mathrm{expr}}^\eta$	$1.00^{+0.16}_{-0.12}$	$1.01^{+0.11}_{-0.11}$	0.03	0.86

Figure S2—1. Estimates of the parameters of linear regressions in human-iPSC data for (**A,B**) genetic noise change, and (**C,D**) gene expression change. Related to ***Figure 2***. (**B,D**) Numbers of included genes with at least 10 cell populations and successful regression, medians and quartile ranges, and F-statistics and p-values of the one-way ANOVA tests for each parameter are given. (**E**) Estimated parameters of the genetic noise change due to differentiation ($\xi^\eta_{d,\mathrm{diff}}$) and differential expression ($\xi^\eta_{d,\mathrm{expr}}$) are given for a subset of N' genes.

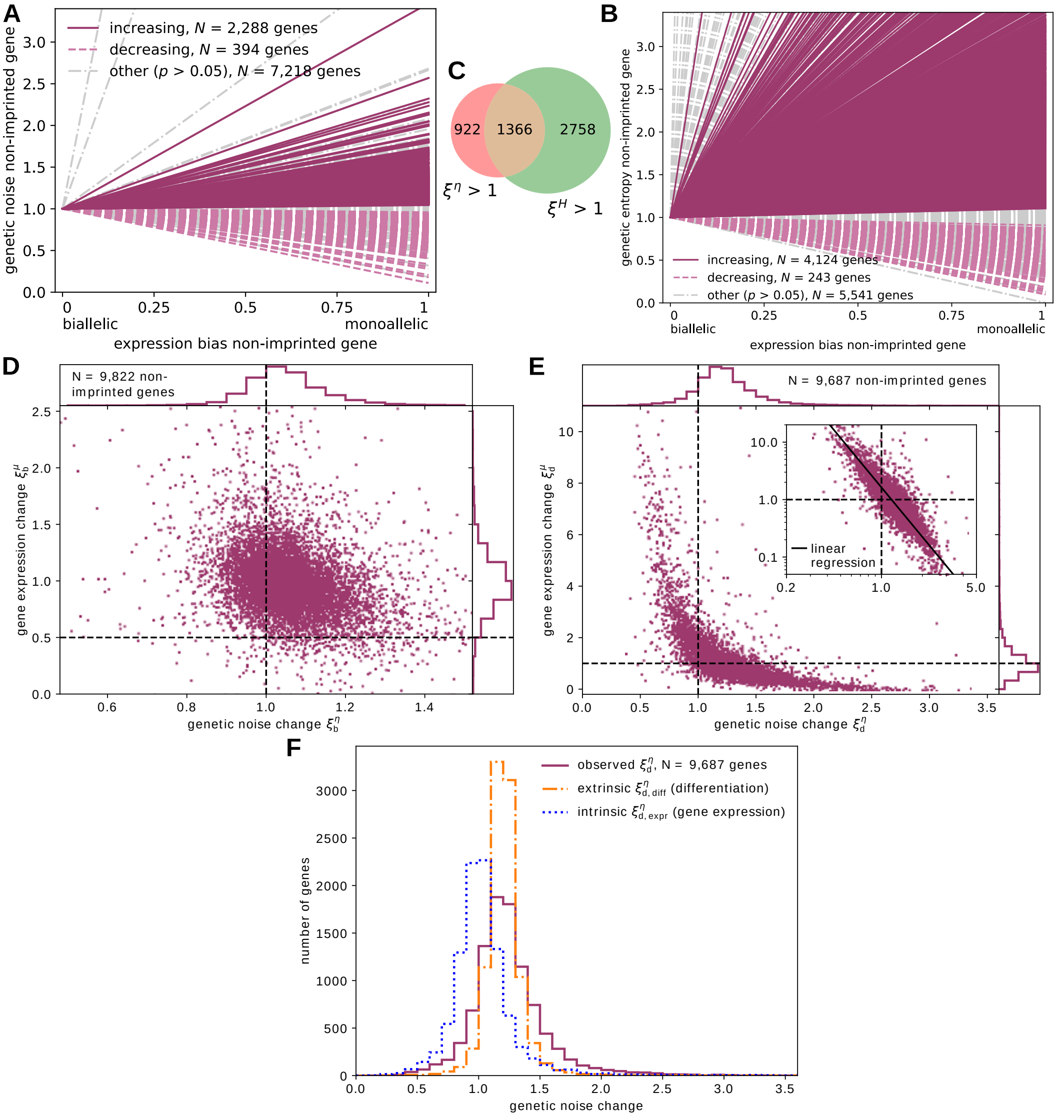

Figure S2—2. Genetic noise, genetic entropy and gene expression of non-imprinted genes in human-iPSC data. (**A**) Normalized genetic noise of 9,900 non-imprinted genes with at least 10 cell populations in dependency of their expression bias, estimated by linear regressions. Of these, 2,288 have an increase ($p < 0.05$) in genetic noise and 394 have a decrease ($p < 0.05$). The mean genetic noise change is $\bar{\xi}_b^\eta = 1.06$ with a standard deviation of $\sigma(\xi_b^\eta) = 0.29$. Related to ***Figure 2***. (**B**) Normalized genetic entropy of 9,908 non-imprinted genes with at least 10 cell populations in dependency of their expression bias, estimated by linear regressions. Related to ***Figure 2***. (**C**) Venn diagram of non-imprinted genes with increase in genetic noise (left) and genetic entropy (right). (**D**) Estimates of gene expression change ξ_b^μ and of genetic noise change ξ_b^η and their estimated uncertainties for 9,822 non-imprinted genes (with at least 10 cell populations and for which the linear regression succeeded). Related to ***Figure 2—6***. (**E**) Estimates of gene expression change ξ_d^μ and of genetic noise change ξ_d^η from day 0 to day 3 of differentiation for 9,687 non-imprinted genes. The observed mean genetic noise change is $\bar{\xi}_d^\eta = 1.26$ with a standard derivation of $\sigma(\xi_d^\eta) = 0.97$ and the mean gene expression change is $\bar{\xi}_d^\mu = 1.36$ with a standard deviation $\sigma(\xi_d^\mu) = 3.21$. The inset depicts the linear regression, which we performed on the values including their uncertainties. The parameters of this linear regression are $\kappa = 2.86 \pm 0.02$ and $\beta = 1.185 \pm 0.001$. Related to ***Figure 2—8A***. (**F**) Distributions of the estimates for extrinsic and intrinsic genetic noise change due to differentiation and due to differential gene expression, respectively. The mean extrinsic genetic noise change is $\bar{\xi}_{d,\mathrm{diff}}^\eta = 1.22$ with a standard derivation of $\sigma(\xi_{d,\mathrm{diff}}^\eta) = 0.83$ and the mean intrinsic genetic noise change is $\bar{\xi}_{d,\mathrm{expr}}^\eta = 1.04$ with a standard derivation of $\sigma(\xi_{d,\mathrm{expr}}^\eta) = 0.31$. Related to ***Figure 2—8B***.

A

	imprinted		non-imprinted		co-expressed of increasing imp.		co-expressed of all imprinted	
total	115		11,116		3,407		10,695	
successful regression	108	100%	9,900	100%	3,407	100%	10,694	100%
increasing ($\xi_b^\eta > 1$)	84	78%	7,029	71%	2,673	78%	7,798	73%
increasing, $p < 0.05$	31	28%	2,288	23%	1,196	35%	2,334	22%
decreasing ($\xi_b^\eta < 1$)	24	22%	2,871	29%	734	22%	2,896	27%
decreasing, $p < 0.05$	6	6%	394	4%	73	2%	330	3%

B

	imprinted		non-imprinted	
total	115		11,116	
successful regression	104	100%	9,829	100%
upregulated ($\xi_b^\mu > 0.5$)	98	94%	9,508	97%
upregulated, $p < 0.05$	77	74%	8,317	85%
downregulated ($\xi_b^\mu < 0.5$)	6	6%	321	3%
downregulated, $p < 0.05$	0	0%	0	0%

Figure S2—3. Statistics of linear regressions for (**A**) genetic noise change and (**B**) gene expression change from bi- to monoallelic expression in human-iPSC data. Related to ***Figure 2***. (**A**) Numbers and percentages of imprinted and non-imprinted genes, and pairs of co-expressed and imprinted genes. For example, 108 of 115 imprinted genes have successful regression and of them, 84 (31 with $p < 0.05$) have increased and 24 (6 with $p < 0.05$) have decreased genetic noise at monoallelic expression. (**B**) Numbers and percentages of imprinted and non-imprinted genes with successful regression for gene expression change, and of genes with up- or downregulation at monoallelic expression.

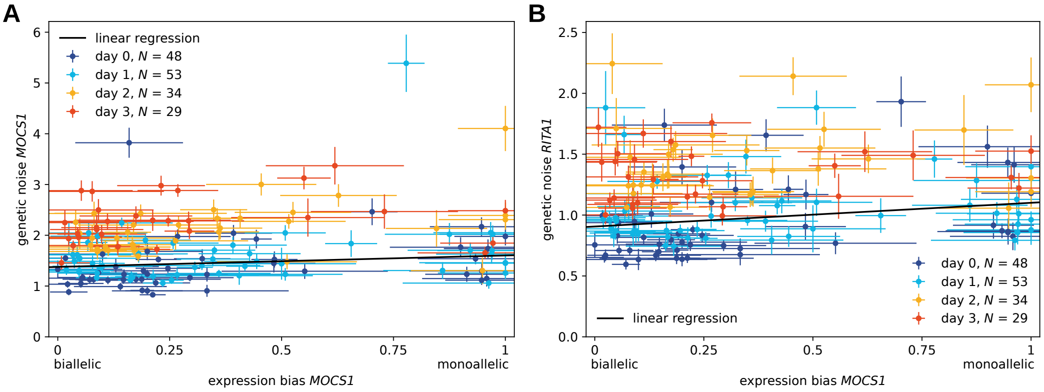

Figure S2—4. Examples of genetic noise in dependency of expression bias and day of differentiation in human-iPSC data. (**A**) Genetic noise of *MOCS1* in dependency of its expression bias and of the day of differentiation. The genetic noise increases both from bi- to monoallelic expression and from day 0 to day 3 of differentiation. The linear regression yields $\xi_b^\eta = 1.16 \pm 0.07$ and $\xi_d^\eta = 1.83 \pm 0.09$. Related to ***Figure 2E***. (**B**) Genetic noise of *RITA1* in dependency of the expression bias of *MOCS1* and the day of differentiation. The *RITA1* genetic noise increases both from bi- to monoallelic *MOCS1* expression and from day 0 to day 3 of differentiation. The linear regression yields $\xi_b^\eta = 1.22 \pm 0.07$ and $\xi_d^\eta = 1.68 \pm 0.07$. Related to ***Figure 2B***.

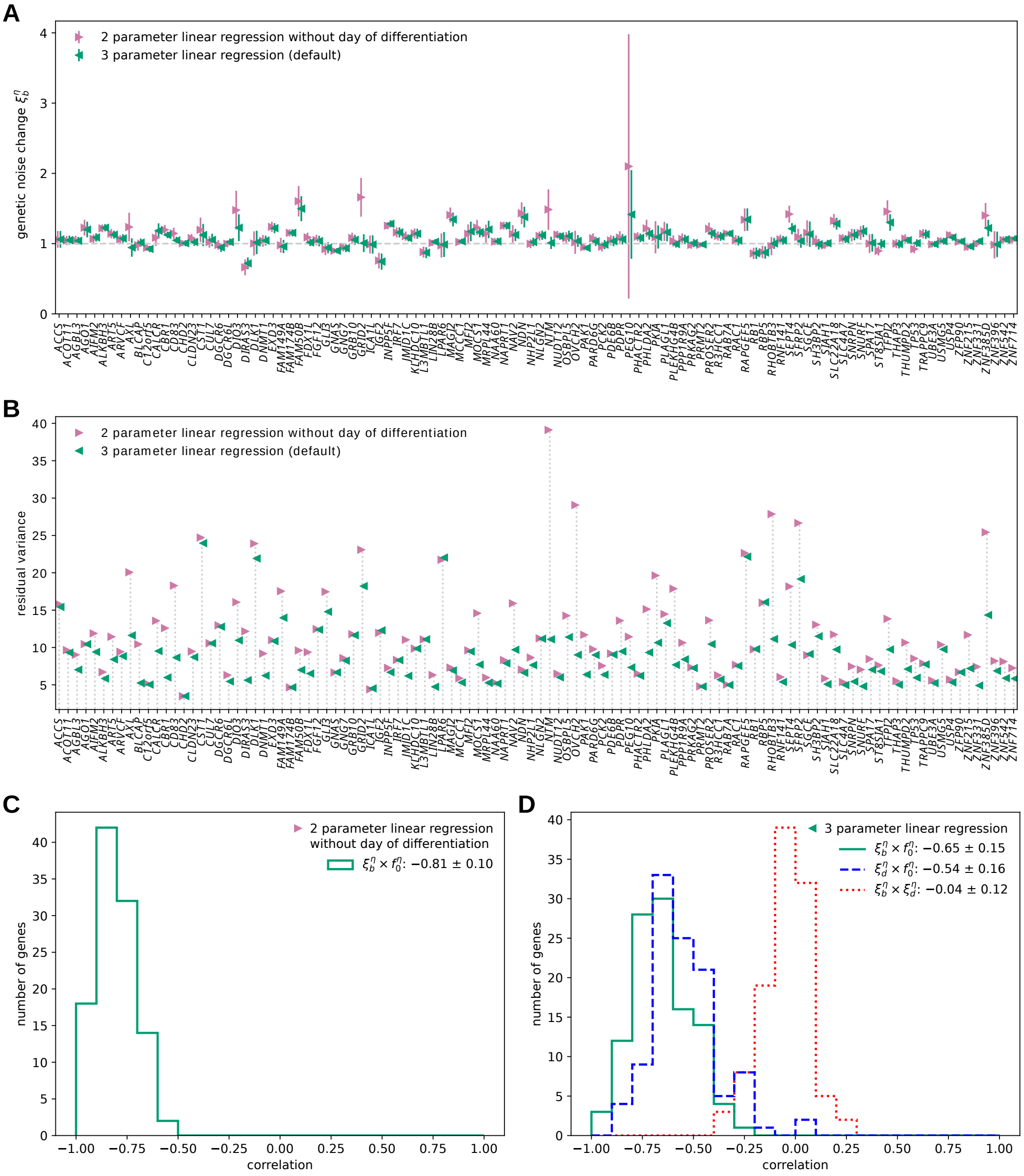

Figure S2—5. Comparison of linear regression of the genetic noise of 108 imprinted genes depending only on the expression bias and depending both on expression bias and day of differentiation in human-iPSC data. Related to ***Figure 2***. (**A**) Genetic noise change from bi- to monoallelic expression ξ_b^η. No difference has been found, and estimated uncertainties are generally smaller or the same for the regression depending on both expression bias and day of differentiation. (**B**) Residual variance, a measure of goodness of fit. It is generally decreased or the same for the regression depending on both expression bias and day of differentiation. (**C**) Distribution of correlation of the parameters of regression depending only on the expression bias. (**D**) Distribution of correlation of the parameters of regression depending both on expression bias and day of differentiation. The estimates of genetic noise change from bi- to monoallelic expression ξ_b^η and from day 0 to day 3 of differentiation ξ_d^η are almost uncorrelated with each other. The genetic noise value at biallelic expression and day 0, f_0^η, is strongly correlated with both estimates of genetic noise change.

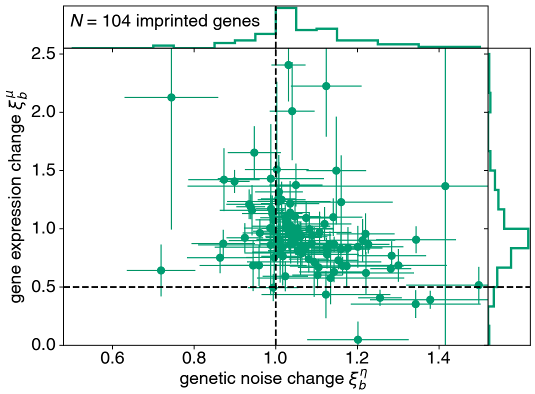

Figure S2—6. Dosage compensation for imprinted genes at monoallelic expression in human-iPSC data. Estimates of gene expression change (ξ_b^μ) and of genetic noise change (ξ_b^η) and their estimated uncertainties for 104 imprinted genes with at least 10 cell populations and succeeded linear regression.
See ***Figure 2—2B*** for corresponding distribution for non-imprinted genes.

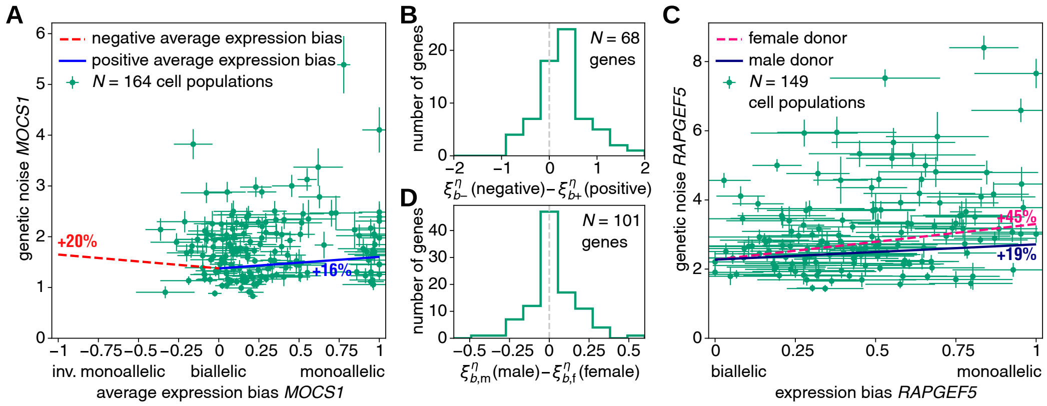

Figure S2—7. Impact of parent of origin and sex of the donor on the genetic noise change in human-iPSC data. (**A**) Genetic noise of *MOCS1* in dependency of its average expression bias. (**B**) Distribution of differences in genetic noise change of the estimates for negative and positive average expression bias. Genes are included if they have at least 10 cell populations with negative average expression bias. (**C**) Genetic noise of *RAPGEF5* in dependency of its expression bias, split by sex of the donor. (**D**) Distribution of differences in genetic noise change of the estimates for male and female donors. Genes are included if they have at least 10 cell populations from both male and female donors.

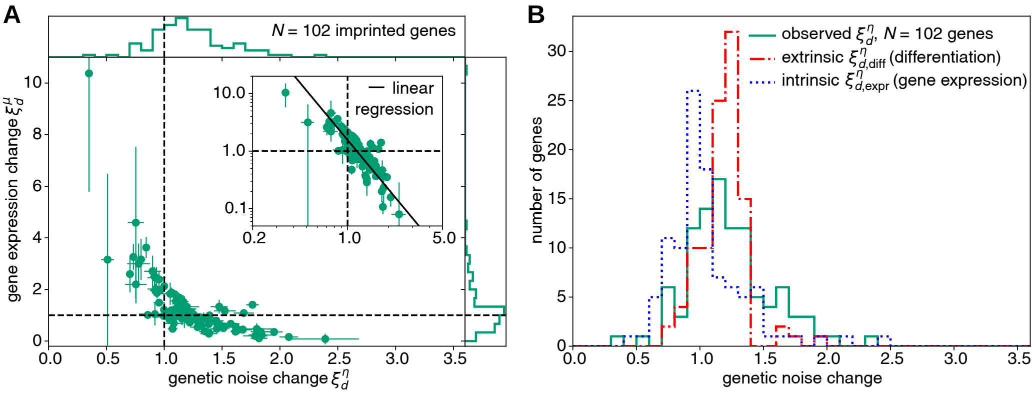

Figure S2—8. Impact of the differentiation on the genetic noise of imprinted genes in human-iPSC data. (**A**) Estimates of gene expression change (ξ_d^μ) and of genetic noise change (ξ_d^η) from day 0 to 3 of differentiation and their estimated uncertainties for 102 imprinted genes with at least 10 cell populations. The dashed lines show the values of no change for $\xi_d^\eta = 1$ and $\xi_d^\mu = 1$. The inset depicts a subset of the same data in logarithmic scale for both axes together with a linear regression. (**B**) Distributions of the estimates for extrinsic and intrinsic genetic noise change due to differentiation and due to differential gene expression, respectively.
See ***Figure 2*—*2C,D*** for corresponding distributions for non-imprinted genes.

A

	human iPSC Cuomo, 2020	mouse ESC Lentini, 2022	mouse embryo Borensztein, 2017	mouse brain Laukoter, 2020
genes included	10,343	12,934	7,773	9,745
cell populations per gene	≤ 408	≤ 38	≤ 11	≤ 7[(A)]; ≤ 8[(N)]
cells per cell population	67 ± 56	90 ± 5	7 ± 4	25 ± 18

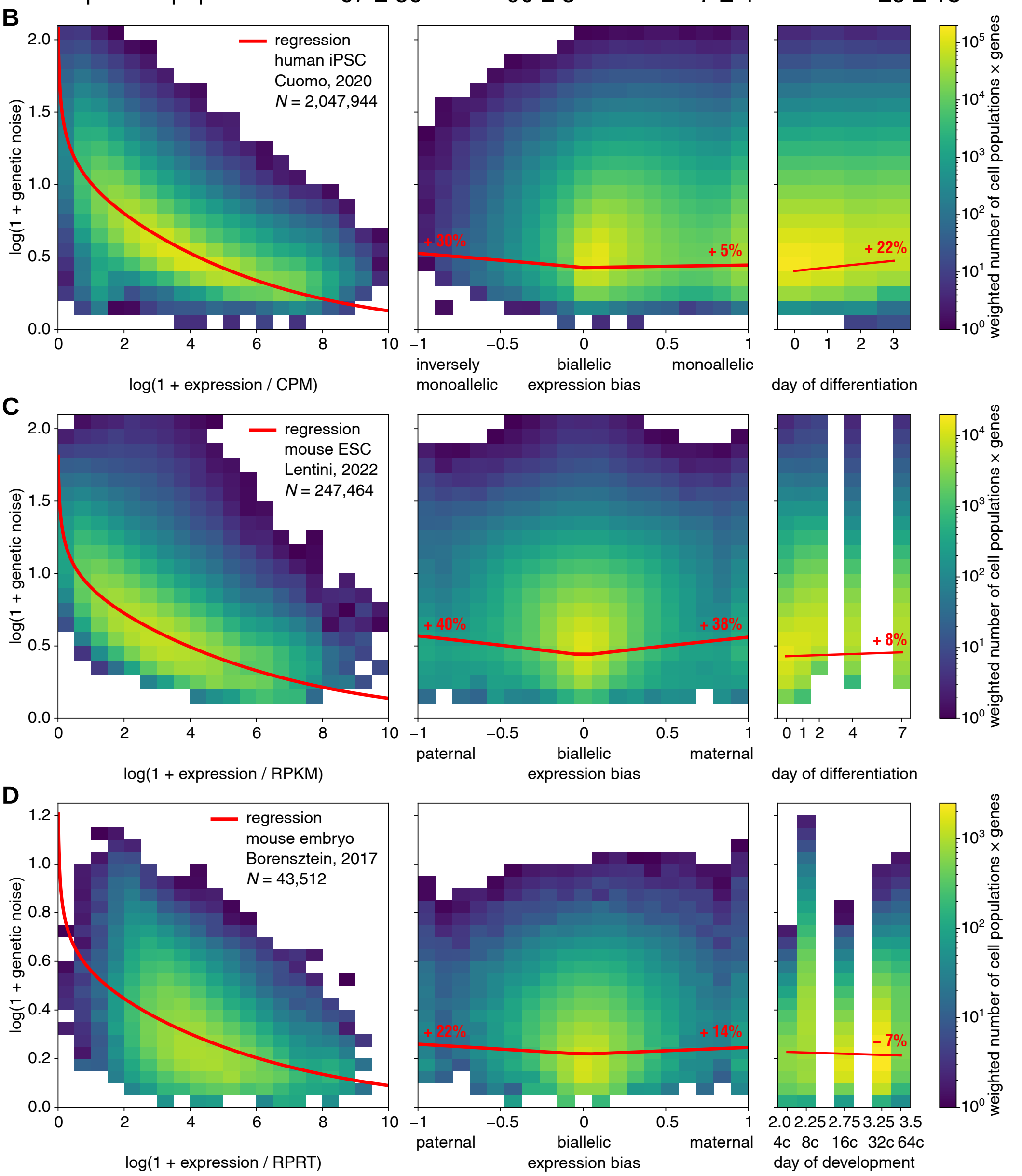

Figure S2—9. Overall analysis of genetic noise increase at monoallelic expression in different datasets. (**A**) Statistics of included genes and cell populations; [(A)] astrocytes, [(N)] neurons. (**B–D**) Genetic noise in dependency of the gene expression, the expression bias and the day of differentiation or development. Each histogram entry corresponds to one cell population and one gene, weighted by the inverse variance of the genetic noise. The regression is shown by the solid line. (**B**) Human iPSC differentiation. (**C**) Mouse ESC differentiation. (**D**) Mouse embryonic development.

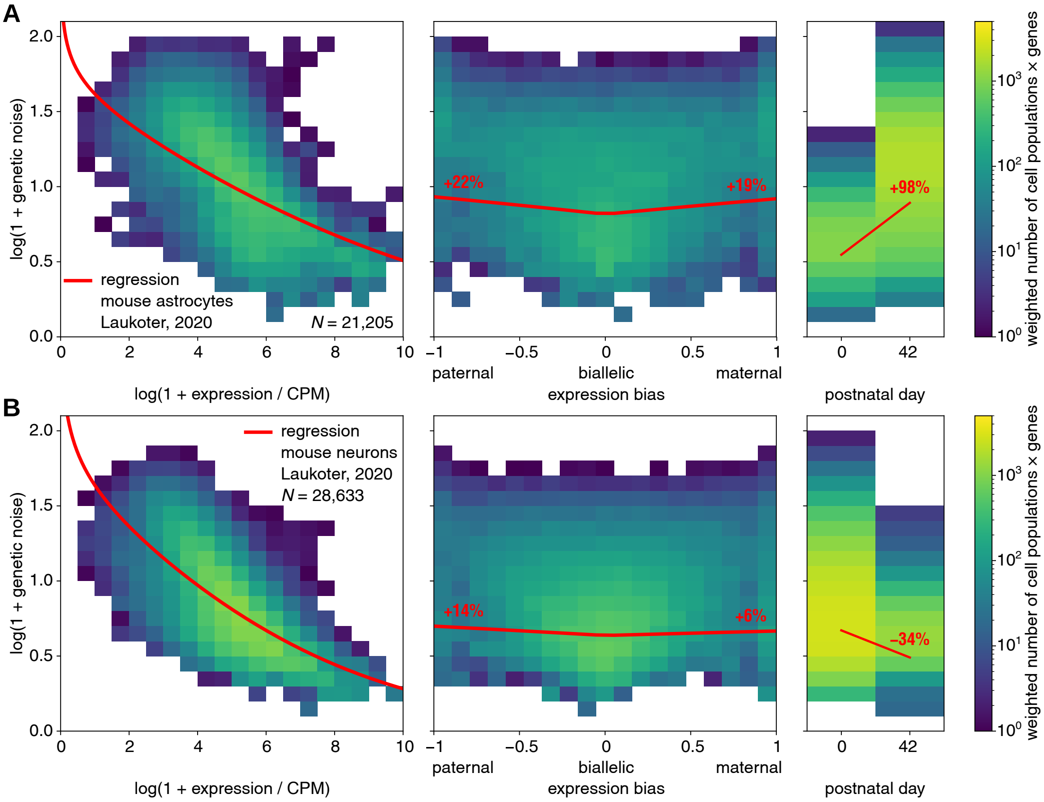

Figure S2—10. Overall analysis of genetic noise increase at monoallelic expression in the mouse brain development for (**A**) astrocytes and (**B**) neurons.

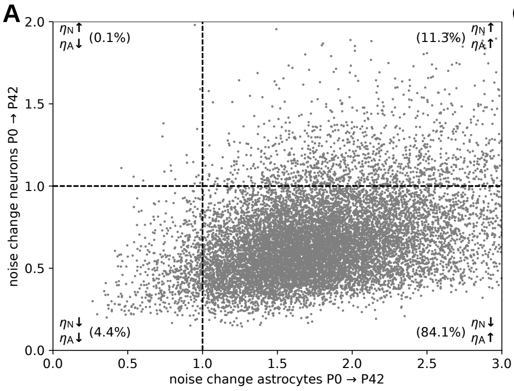

B

	genetic noise change P0 → P42 astrocytes $\xi^{\eta}_{d,A}$	neurons $\xi^{\eta}_{d,N}$	number of genes
$\eta_N\downarrow$ $\eta_A\downarrow$	< 1	< 1	642 (4.4%)
$\eta_N\uparrow$ $\eta_A\downarrow$	< 1	>1	21 (0.1%)
$\eta_N\downarrow$ $\eta_A\uparrow$	> 2	< 1	3,530 (24.4%)
$\eta_N\uparrow$ $\eta_A\uparrow$	> 1	>1	1,638 (11.3%)

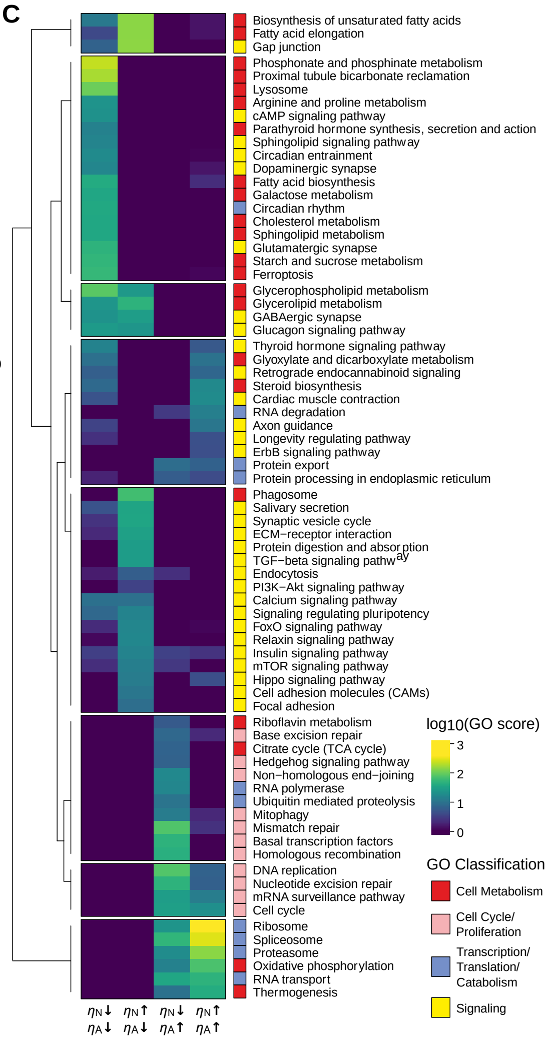

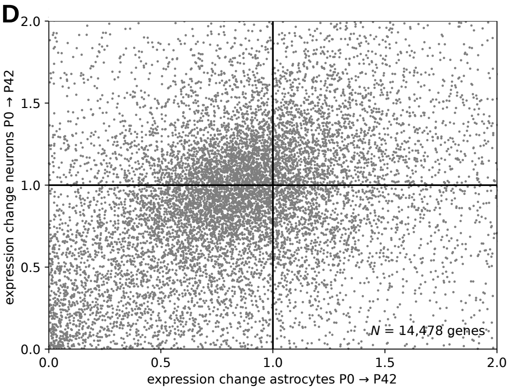

Figure S2—11. Gene ontology of genes with genetic noise increase or decrease from P0 to P42 in astrocytes and neurons. (**A**) Genetic noise change from P0 to P42 in astrocytes and neurons. The genetic noise of the majority of 84.1% genes increases in astrocytes ($\eta_A\uparrow$) while it decreases in neurons ($\eta_N\downarrow$). (**B**) Classification of genes according to genetic noise increase or decrease. (**C**) KEGG pathway enrichment on the four groups of genes from (**B**) was analyzed with EnrichR. The GO score consists of the database combined score. (**D**) Gene expression change from P0 to P42 in astrocytes and neurons. Overall, the gene expression changes only slightly.

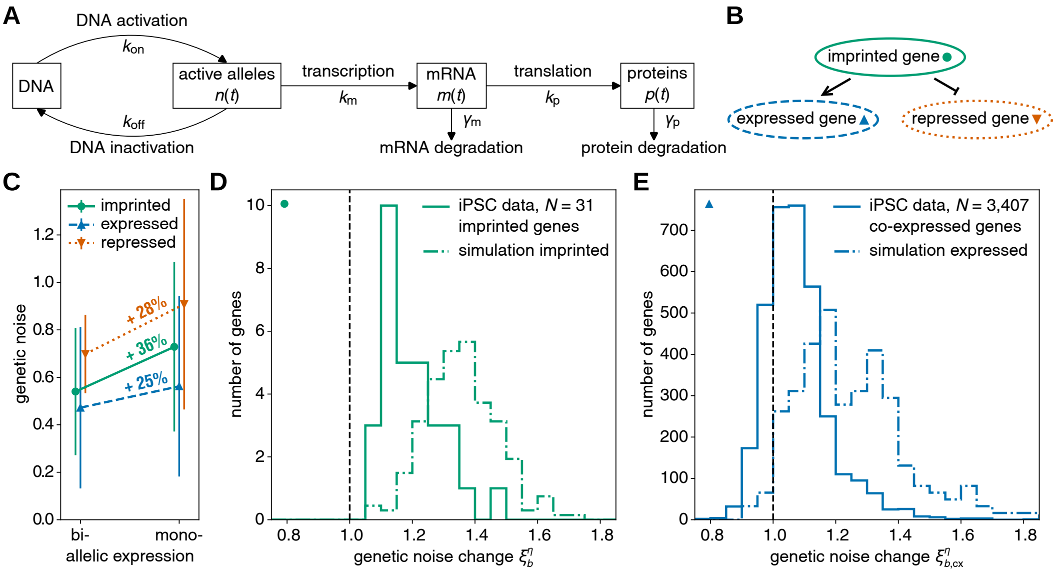

Figure S2—12. Simulated gene regulatory network and genetic noise. (**A**) Processes of gene expression for a coding gene with rate constants. (**B**) Network of three genes with an imprinted gene positively regulating the transcription of one gene and repressing another gene. (**C**) Simulated genetic noise of the three genes in the network in dependency of the expression bias of the imprinted gene. In both bi- and monoallelic expression $N = 208$ simulations have been performed with different rate constants. (**D**) Distributions of genetic noise change of 31 imprinted genes with genetic noise increase observed in human-iPSC data (***Figure 2***), and of simulated imprinted genes. (**E**) Distributions of genetic noise change of co-expressed genes observed from human-iPSC data (**??**), and of simulated expressed genes.

A

		imprinted gene (i)		expressed gene (e)	repressed gene (r)	
maximum number of active alleles	$\tilde{n}$	2	1	2	2	
DNA activation	k_{on}	0.5		$0.02 \cdot p^{i}(t)$	0.5	h^{-1}
DNA inactivation	k_{off}	8		8	$0.15 \cdot p^{i}(t)$	h^{-1}
mRNA transcription	k_m	225	450	150	150	h^{-1}
mRNA degradation	γ_m	0.75		1.5	0.5	h^{-1}
protein translation	k_p	1.25		1.25	1.25	h^{-1}
protein degradation	γ_p	0.35		0.35	0.35	h^{-1}

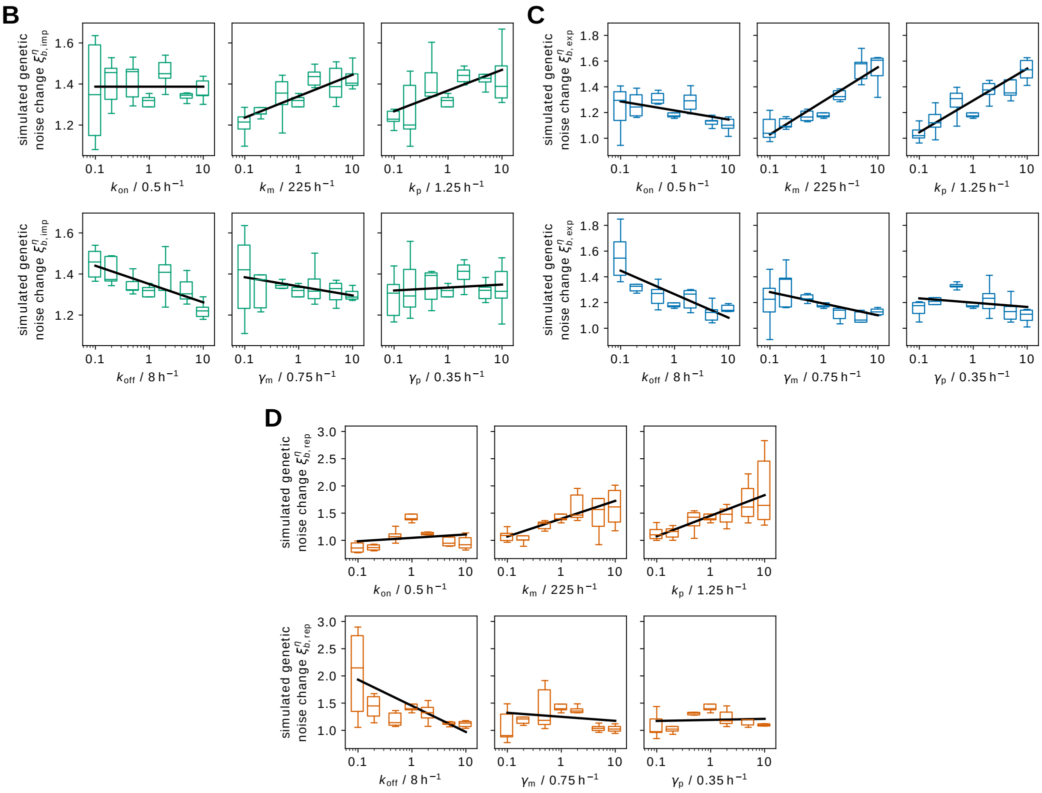

Figure S2—13. Simulated genetic noise change from bi- to monoallelic expression in dependency of gene expression process rates. (**A**) The nominal constants used in the simulation of gene expression of three genes. (**B–D**) In each sub-panel the ratios of simulated genetic noise of $N = 38$ simulations with mono- over $N = 38$ simulations with biallelic expression of the imprinted gene are shown. Each time only one process rate of the expression of the imprinted gene is varied from 0.1 to 10 times the nominal value. (**B**) Genetic noise change of the imprinted gene. (**C**) Genetic noise change of the expressed gene. (**D**) Genetic noise change of the repressed gene.
