# Supplementary material for: Monoallelic gene expression in developing cells increases genetic noise and Shannon entropy
Source: Commun Biol. 2025 Jun 4;8:857. doi: 10.1038/s42003-025-08128-2 (PMC12137721; doi:10.1038/s42003-025-08128-2)
Supplement: Supplementary file 2 — Description of Additional Supplementary Materials [file 42003_2025_8128_MOESM2_ESM.pdf]

Description of Additional Supplementary Files

File name: Supplementary Data 1

Description: The source data behind the figures in the paper

File name: Supplementary Data 2

Description: The regression parameters of genetic noise change from biallelic to monoallelic expression, and from day 0 to day 3 of differentiation for imprinted genes in human-iPSC data

File name: Supplementary Data 3

Description: The regression parameters of genetic entropy change from biallelic to monoallelic expression, and from day 0 to day 3 of differentiation for imprinted genes in human-iPSC data

File name: Supplementary Data 4

Description: The regression parameters of genetic noise change from biallelic to monoallelic expression, and from day 0 to day 3 of differentiation for pairs of imprinted genes and co-expressed genes in human-iPSC data

File name: Supplementary Data 5

Description: The regression parameters of genetic entropy change from biallelic to monoallelic expression, and from day 0 to day 3 of differentiation for pairs of imprinted genes and co-expressed genes in human-iPSC data

File name: Supplementary Data 6

Description: The regression parameters of genetic noise change from biallelic to monoallelic expression, and from day 0 to day 3 of differentiation for non-imprinted genes in human-iPSC data

File name: Supplementary Data 7

Description: The regression parameters of genetic entropy change from biallelic to monoallelic expression, and from day 0 to day 3 of differentiation for non-imprinted genes in human-iPSC data

File name: Supplementary Data 8

Description: The regression parameters of gene expression change from biallelic to monoallelic expression, and from day 0 to day 3 of differentiation for imprinted genes in human-iPSC data

File name: Supplementary Data 9

Description: The regression parameters of gene expression change from biallelic to monoallelic expression, and from day 0 to day 3 of differentiation for non-imprinted genes in human-iPSC data

File name: Supplementary Data 10

Description: The regression parameters of genetic noise change from average expression bias 0 to −1, 0 to +1, and from day 0 to day 3 of differentiation for imprinted genes in human-iPSC data, testing the parent-of-origin effect

File name: Supplementary Data 11

Description: The regression parameters of genetic noise change from biallelic to monoallelic expression of female and male donors, and from day 0 to day 3 of differentiation for imprinted genes in human-iPSC data, testing the sex of the donor
